# WNT inhibitor SP5-mediated SERPING1 suppresses lung adenocarcinoma progression via TSC2/mTOR pathway

**DOI:** 10.1038/s41419-025-07440-3

**Published:** 2025-02-17

**Authors:** Yefeng Shen, Xiaofeng Dong, Xujia Li, Zhiyuan Shi, Tingting Shao, Junlan Jiang, Jian Song

**Affiliations:** 1 Institute of Cardiovascular Sciences, Guangxi Academy of Medical Sciences, Nanning, China; 2 Department of Thoracic Surgery, Beijing Friendship Hospital, Capital Medical University, Beijing, China; 3 Department of Hepatobiliary, Pancreas and Spleen Surgery, The People’s Hospital of Guangxi Zhuang Autonomous Region (Guangxi Academy of Medical Sciences), Nanning, China; 4 State Key Laboratory of Oncology in South China, Guangdong Provincial Clinical Research Center for Cancer, Sun Yat-sen University Cancer Center, Guangzhou, China; 5 School of Pharmaceutical Science and Technology, Faculty of Medicine, Tianjin University, Tianjin, China; 6 Department of Pediatrics, Peking University First Hospital, Beijing, China; 7 Department of Pathology, the First Affiliated Hospital, Anhui Medical University, Hefei, China; 8 Pathology Center, Anhui Medical University, Hefei, China; 9 Department of Radiation Oncology, Renji Hospital, School of Medicine, Shanghai Jiao Tong University, Shanghai, China

**Keywords:** Non-small-cell lung cancer, Tumour-suppressor proteins

## Abstract

The long-term outlook for patients grappling with lung cancer (LC) remains bleak, with lung adenocarcinoma (LUAD) emerging as the most predominant histological subtype. Our Mendelian randomization (MR) investigation spotlighted that heightened levels of the circulating protein serpin peptidase inhibitor family G1 (SERPING1) substantially mitigated LC risk. The fusion of multi-omics strategies unveiled that SERPING1 exhibited diminished expression in LUAD patients compared to healthy individuals both in tissues and serum, with LUAD individuals showcasing elevated SERPING1 expression demonstrating improved prognoses. Furthermore, SERPING1 expression exhibited a robust correlation with the efficacy of immunotherapy. Through meticulous in vivo and in vitro analyses, we unraveled that SERPING1 impeded the proliferation, migration, invasion and wound healing of LUAD cells via the tuberous sclerosis 2 (TSC2)/mammalian target of rapamycin (mTOR) pathway. Mechanistically, WNT inhibitor- Specificity Protein (SP5) was delineated as facilitator of SERPING1 transcription by binding to the SERPING1 gene promoter. Intriguingly, aside from the association between SERPING1 and systolic blood pressure, glycosylated hemoglobin (HbA1c), type I diabetes, no discernible link between SERPING1 overexpression and heightened risks of other cardiometabolic conditions and diseases was evident. In summary, SERPING1 emerges as a novel tumor suppressor gene and SP5/SERPING1/TSC2 is a promising therapeutic target in the context of LUAD.

## Introduction

Lung cancer (LC) stands out for the high incidence and mortality rates, contributing to a staggering 2.2 million new cases and 1.79 million deaths globally each year [1]. This malignancy encompasses non-small cell lung cancer (NSCLC) [2] and small cell lung cancer (SCLC) [3], with NSCLC and SCLC constituting 85% and 15% of diagnoses, respectively. Within NSCLC, lung adenocarcinoma (LUAD) reigns as the most prevalent histological subtype. Despite significant therapeutic strides in LC management encompassing surgery, radiation, chemotherapy, targeted therapies addressing oncogenic drivers, and immunotherapy, the long-term survival rates for LUAD patients remain discouraging [4]. This underscores the critical imperative to explore viable, easily accessible biomarkers for the prognosis of LC that could be double as therapeutic targets.

Mendelian randomization (MR) emerges as a favored method for delineating causality, wielding substantial efficacy in drug target validation [5–7]. Concurrently, MR serves as a potent analytical tool for assessing the safety of genetic inhibitors [8–10]. In a bid to unearth therapeutic avenues for aortic aneurysms, proteasome 20S subunit alpha 4 (PSMA4) and plasminogen activator urokinase (PLAU) were pinpointed to colocalize with aortic aneurysms through MR analysis, suggesting that targeting PLAU and PSMA4 inhibition might curtail the risk of aortic aneurysms [11]. Fusing MR with omics methodologies spanning RNA sequencing, single cell RNA sequencing (scRNA-seq), and proteome has proven instrumental in exhaustively identifying oncogenic or tumor-suppressor genes, unraveling tumorigenesis nuances, and decoding tumor progression [12, 13]. The application of omics approaches has notably enhanced the precision in unearthing potential drug targets [14, 15]. Nonetheless, the realm of integrated omics-wide analyses for novel therapeutic targets in LC remains largely uncharted.

Serpin peptidase inhibitor family G1 (SERPING1) was identified as a novel marker in diagnosis and prognostic prediction in prostate cancer, indicating a strong correlation between decreased expression and the poor prognosis [16]. However, whether SERPING1 plays vital roles in LUAD remains unknown. Meanwhile, the tuberous sclerosis 2 (TSC2)/mammalian target of rapamycin (mTOR) pathway was found to be involved in the tumorigenesis [17] and autophagy [18], while the association between SERPING1 and the TSC2/mTOR pathway is not discovered.

This study embarks on a proteome-wide MR exploration by combining human plasma proteomes with genomic data to methodically delineate circulating protein biomarkers implicated in elevating LC risk. Moreover, a myriad of dimensions including bulk transcriptome profiling, single-cell transcriptome analysis, spatial transcriptome, and rigorous in vitro and in vivo experiments were harnessed to identify and validate the functional roles of the target protein and potential mechanisms in LUAD. Crosstalk between mTOR and WNT/β-catenin signaling pathway was investigated. The safety of the therapeutic targets was emphasized on the basis of the MR methodology. Cumulatively, the data converge to spotlight SERPING1 as a novel tumor suppressor gene in the context of LUAD.

## Methods

### MR analysis overview

We employed a robust two-sample MR strategy to identify circulating proteins associated with LC risk. Additionally, we meticulously evaluated the safety profiles and potential alternative indications of these circulating target proteins. Notably, our study adhered to the guidelines outlined in the STROBE-MR statement.

### Data sources

Our study drew data for from several key sources. Initially, protein quantitative trait loci (pQTL) genome-wide association study (GWAS) data, crucial for identifying potential targets as disease exposures, were sourced from the INTERVAL cohort and the KORA F4 cohort. Furthermore, summary data concerning LC and its subtypes as disease outcomes primarily originated from the ILCCO cohort and the FinnGen database. To delve into the safety aspects and alternative indications for potential targets, we selected data on 17 cardiometabolic traits and 11 cardiovascular diseases from the UKB database (Neale lab and MRC-IEU) and various other extensive cohorts (including the GLGC Cohort, MAGIC Cohort, GIANT Cohort, MEGASTROKE Cohort, HERMES Cohort, DIAGRAM Cohort, GERA Cohort, and CKDGen Cohort). For a comprehensive understanding of each cohort, detailed descriptions can be found in Table S1.

### Mendelian randomization

MR hinges on three main assumptions. Firstly, instrumental variables (IVs) should exhibit robust associations with the exposure under scrutiny. Second, these IVs must remain unaffected by known or unknown confounders. Finally, IVs should solely influence outcomes through the exposure being studied. Through large-scale GWAS of circulating proteins, it has been revealed that the genetic determinants of these proteins predominantly lie cis to the encoding genes. Leveraging MR with cis-acting single nucleotide polymorphisms (SNPs) serves to mitigate potential horizontal pleiotropy while bolstering the validity of MR assumptions. Cis-SNPs, closely correlated with proteins, likely contribute directly to gene transcription, thereby impacting circulating protein levels. We meticulously selected independent (r^2^ ≤ 0.001) cis-pQTL SNPs from the two pQTL GWAS datasets that exhibited significant associations with circulating proteins (*p* < 5×10^−8^).

For proteins linked to a single cis-SNP, we employed the Wald ratio method to estimate the protein’s impact on the risk of LC. In cases where multiple cis-SNPs were involved, we predominantly utilized the inverse variance weighted (IVW) method to derive effect estimates [19]. To account for the detection of multiple proteins, the Benjamini-Hochberg correction was applied, although it may be conservative given that partial correlations among some proteins. The Mendelian randomization analysis mentioned above was executed using the TwoSampleMR R package [20]. Since most proteins are associated with only one cis-SNP, assessments for heterogeneity and horizontal pleiotropy tests were unavailable in this context.

### Colocalization analysis

To validate the reliability of candidate proteins influencing LC as supported by MR, we conducted colocalization analyses of proteins within the INTERVAL cohort using the coloc R package. Colocalization analysis serves as a metric to assess the posterior probability, determining whether two GWAS signatures (in this instance, protein levels and LC) are influenced by the same genetic variant or if they represent distinct causal variants in linkage disequilibrium with each other. Locus Zoom plots were generated to visually represent the outcomes of the colocalization analyses [21].

### Bioinformatics analysis

Employing a range of bioinformatics tools, including bulk transcriptomes, single-cell transcriptomes, and spatial transcriptomes, we delved into the differential expression patterns, functional enrichments, immune microenvironment characteristics, and predictive capabilities (diagnostic, prognostic, and immunotherapeutic) of the potential target gene. The upstream transcription factors of SERPING1 and their potential binding sites were predicted using the JASPAR database (http://jaspar.genereg.net/).

### Data sources

Bulk transcriptome data were sourced from various datasets within the Cancer Genome Atlas (TCGA) and GEO databases, encompassing multiple LC datasets and immunotherapy datasets across various tumor types. Additionally, single-cell transcriptome data were retrieved from 10.24433/CO.0121060.v1. Information regarding the spatial transcriptome of LC was obtained from the Single-cell and Spatially-Resolved Cancer Resources (SCAR) database (http://scaratlas.com) for the LC spatial transcriptome. Detailed descriptions of each dataset are provided in Table S2 and Table S3.

### Single-cell RNA sequencing (scRNA-seq)

The scRNA-seq data were analyzed using the Seurat package (version 4.2). Cells with gene counts below 300 or exceeding 10,000, or with more than 20% mitochondrial genes, were filtered out. Cell-type markers were sourced from previous studies (19, 20). Subsequently, 26,286 cells from tumor tissues and adjacent normal tissues were retained for further analysis. Uniform manifold approximation and projection (UMAPs) were constructed using principal components. Cells were arranged along a branched pseudotime trajectory using Monocle 2/3 (v0.1.3) (21), and the “plot_pseudotime_heatmap” function was utilized to visualize heatmaps of dysregulated genes along the pseudotime trajectories.

### RNA sequencing (RNA-seq)

After SERPING1 overexpression in A549 cells, total RNA was extracted and used for sequencing library construction. RNA-seq was conducted on the NovaSeq 6000 (Illumina) platform by Nobelio Biotechnology (Guangzhou, China). (https://david.ncifcrf.gov/). Differential expression analyses were conducted between the two groups with the limma package, and the filter condition for differentially expressed genes (DEGs) was set to |log2Foldchange | >1 and the adjusted *p* < 0.05. Kyoto Encyclopedia of Genes and Genomes (KEGG) pathway analysis was executed for all DEGs to investigate whether these genes were enriched in the biological processes.

### Immune infiltration analysis

The immune score, stromal score, and estimate score stand for immune infiltration and stromal cell levels [22]. These data were obtained by applying calculations to the raw matrix with the Estimate package (https://bioinformatics.mdanderson.org/estimate/index.html). Moreover, a wide range of deconvolution tools, including xCEll, CIBERSORT, Timer, Quanti-Seq, EPIC, and MCP-counter, were deployed to screen for diverse types of immune cells in the tumor tissues. The Timer2.0 website (http://timer.comp-genomics.org/) and immunedeconv package offered available access to and acquisition of these data [23].

### Prediction of immunotherapy response

Multiple considerations were required to assess the predictive function of the target gene in the response to immunotherapy. First, since the Immune Phenotype Score (IPS) was a valid predictor of immunotherapy response to targeted CTLA-4 and PD-1, the IPS scores (https://tcia.at/home) were obtained to characterize the immunogenicity and immunoreactivity of the tumors. Second, the correlation between the expression of immune checkpoints, MHC molecules, and the expression of the target gene was assessed. Finally, several immunotherapy datasets were used to investigate the value of the target gene for predicting response to immunotherapy.

### Cell culture, plasmid transfection, and lentivirus infection

LUAD cells A549, H1975, PC9, H1299, and bronchial epithelial cells BEAS-2B were purchased from the American Type Culture Collection (ATCC). Cells were cultured in Dulbecco’s modified Eagle’s medium (DMEM, Gibco, Grand Island, NY, USA) supplemented with 10% fetal bovine serum (FBS; Gibco) at 37 °C in a humidified chamber with 5% CO_2_. Cells were treated with 10 μM MHY1485 (MCE, USA) for 24 h. Human recombinant SERPING1 and supernatant from SERPING1-overexpressed cells were used to treat cells. Plasmids and lentiviruses that overexpress target genes were established by the Medical Discovery Leaser (MDL) company. Cells were transfected with plasmids or infected with lentiviruses according to the manufacturer’s instructions. SiRNAs used were used in Table S4.

### Enzyme-linked immunosorbent assay (ELISA)

Human plasma was collected from the People’s Hospital of Guangxi Zhuang Autonomous Region and the collection of human plasma was approved by the Life Ethics Committees of the People’s Hospital of Guangxi Zhuang Autonomous Region. Levels of SERPING1 in human plasma were measured by using the Human SERPING1 ELISA Kit (cat. no. KIT10995, Sino Biological, China) following the manufacturer’s protocol.

### RNA isolation and quantitative real-time polymerase chain reaction (qPCR) analysis

Total RNA was extracted from cells using the TRIzol reagent (Invitrogen, Grand Island, NY) before synthesis of cDNA via the TaqMan Reverse Transcription Kit (Thermo Fisher Scientific, Waltham, MA, USA). Next, qPCR analysis was performed using a SYBR Green kit (Roche, Mannheim, Germany) according to the manufacturer’s instructions. The primer sequences were as follows: β-actin, Forward 5′- TCCTCCTGAGCGCAAGTACTCC -3′, Reverse 5′- CATACTCCTGCTTGCTGATCCAC -3′; SERPING1, Forward 5′- ACTTGGAGCTCATCAACACC -3′, Reverse 5′- GTTGTCTTCCACTTGGCACT -3′. TSC2, Forward 5′- GCTGAACATCATCGAACGGC -3′, Reverse 5′- CGTGGAACTCGTTCTGGTCA -3′. Specificity Protein (SP5), Forward 5′- CCTACAAAGAGGCCTGGTGT -3′, Reverse 5′- CTCTGGTACTGCGCAAAGTC -3′. The relative expression of each gene was calculated by the 2 − ΔΔCt method.

### Western blot

We extracted total protein from cells and quantified protein concentration using a bicinchoninic acid kit (BCA, Beyotime Biotechnology, Beijing, China). The proteins were separated by 10% SDS-PAGE gels and transferred to a polyvinylidene difluoride (PVDF) membrane. After it was blocked with 10% nonfat milk in PBST for 1 h at room temperature, the membrane was incubated with primary antibodies (SERPING1, 1:1000, cat. no. DF4299, Affinit, China; β-actin, 1:3000, cat. no. AF7018, Affinit, China; TSC2, 1:1000, cat. no. AF6334, Affinit, China; mTOR, 1:1000, cat. no. AF6308, Affinit, China; p-mTOR, 1:1000, cat. no. AF3308, Affinit, China; 4E-BP1, 1:1000, cat. no. AF6432, Affinit, China; p-4E-BP1, 1:1000, cat. no. AF3830, Affinit, China; SP5, 1:1000, cat. no. ab36593, Abcam, Cambridge, UK) overnight at 4 °C and treated for 1 h with secondary antibodies at room temperature.

### Immunohistochemistry (IHC)

Formalin-fixed LUAD and paired adjacent normal tissues were sectioned, and paraffin sections were cut into 5 µm sections and mounted on slides. After deparaffinization and rehydration, we conducted antigen retrieval and incubated the sections with primary antibodies against respective proteins: SERPING1 (1:100, cat. no. 12259-1-AP, Proteintech, USA), Ki67 (1:500, cat. no. 28074-1-AP, Proteintech, USA), and Collagen I (1:500, cat. no. 67288-1-Ig, Proteintech, USA). This was followed by incubation with secondary antibodies for 2 h at room temperature. The optical density from pathological images was quantitatively analyzed by Image-Pro Plus (IPP) 6.0 software (Media Cybernetics Inc., USA).

### Immunofluorescence analysis (IF)

Tissues were fixed in 4% paraformaldehyde for 15 min and permeabilized in 0.2% Triton X-100 for 5 min. Subsequently, the slides were incubated with primary antibodies (SERPING1, 1:200, cat. no. 12259-1-AP, Proteintech, USA) at 4 °C overnight and then with a secondary antibody (green) in the dark. Nuclei were stained with DAPI at room temperature for 5 min. The images were captured using a laser-scanning confocal microscope (LSM-710, Zeiss, Germany).

### Cell proliferation, transwell migration, Matrigel invasion, and wound healing assays

We assessed cell proliferation using the cell counting kit-8 (CCK-8) (Dojindo, Kumamoto, Japan). Cells were seeded into 96-well plates and 10 µl of CCK-8 solution was added to each well. We measured the absorbance at 450 nm using a microplate reader. For migration and invasion assays, cells suspended in the serum-free medium were seeded onto the upper chamber, and a medium supplemented with 10% FBS was added in the lower chamber, coated with for invasion or without Matrigel for migration. After 48 h of incubation, we removed cells from the upper chamber and fixed the migrated or invaded cells with 4% paraformaldehyde for 10 min. Subsequently, we stained them with 0.5% crystal violet. Wound healing was assessed by seeding cells onto six-well plates and wounds were made using sterile pipette tips. We photographed the wound healing process at 0 h, 24 h, and 48 h and quantified the wound area using ImageJ (National Institutes of Health, Bethesda, MD, USA).

### Nude mouse xenograft model

Six-week-old female BALB/c nude mice were purchased from SpePharm (Beijing) Biotechnology Co., Ltd. Company and randomly divided into three groups (*n* = 5 per group). Approximately 5 × 10^6^ cells were resuspended and inoculated subcutaneously into the flanks of nude mice. Tumor length (D), width (d), and body weight were measured every 2 days, and after 31 days of inoculation, the mice were sacrificed. Xenografts were removed and weighed, and tumor volume was calculated using the formula: (D*d2)/2. IHC was performed to investigate the proteins in the xenografts.

### Chromatin immunoprecipitation-quantitative PCR (CHIP-qPCR)

A549 and H1299 cells were harvested and fixed using 1% formaldehyde in PBS for 10 min and the reaction was stopped with 1.25 M glycine for 5 min. The fixed cells were resuspended in a lysis buffer for 1 h. Next, the chromatins were extracted and sonicated to 200–500 bp length. After reserving 2% of the sample as input, the remaining chromatin solution was incubated with the primary antibody of SP5 (cat. no. DF4299, Abcam, Cambridge, UK) or IgG negative control overnight at 4 °C. The mixture was washed and then eluted with DNA release buffer and protease K. The enrichment of SERPING1 was analyzed by qPCR. Primers of SERPING1 used for CHIP assays are as follows. Forward 5’- ACTTGGAGCTCATCAACACC-3’, Reverse 5’- GTTGTCTTCCACTTGGCACT-3’.

### Luciferase reporter assays

The luciferase plasmids containing predicted three binding sites were constructed respectively. A549 and H1299 cells were transfected with luciferase plasmids using Lipofectamine 2000 (11668019, Invitrogen, Carlsbad, CA, USA) according to the manufacturer’s instructions. Cells were lysed and the activities of firefly luciferase and renilla luciferase were measured by using the Luciferase Reporter Gene Assay Kit (Yeasen, China).

### Statistical analysis

All experiments were independently performed in triplicate. Data were presented as mean ± standard deviation (SD). Continuous variables were expressed as mean ± SD or median and interquartile range, and comparisons between groups were accomplished using the Student’s *t*-test or Wilcoxon tests. Categorical variables were reported as counts and percentages, and comparisons between groups were executed using the chi-square test or Fisher exact probability method. A Spearman correlation was conducted to explore the association between the two variables. Cox proportional hazard models were applied to evaluate the prognosis. The “meta” package runs a meta-analysis of all the hazard ratio (HR) values calculated by the Cox model. The Kaplan-Meier method was employed to predict survival, while the significance was determined through the log-rank test. Receiver operating characteristic (ROC) curves were used to estimate the predictive value of genes for diagnosis and efficacy, and the area under the curve (AUC) was calculated as the basis for assessing performance. All statistical analyses were performed using R statistical software (version 4.3.1), and *p* < 0.05 was considered statistically significant. * *p* < 0.05; ** *p* < 0.01; *** *p* < 0.001; **** *p* < 0.0001.

## Results

### SERPING1 exhibited low expression levels in LUAD

**Fig. 1:**
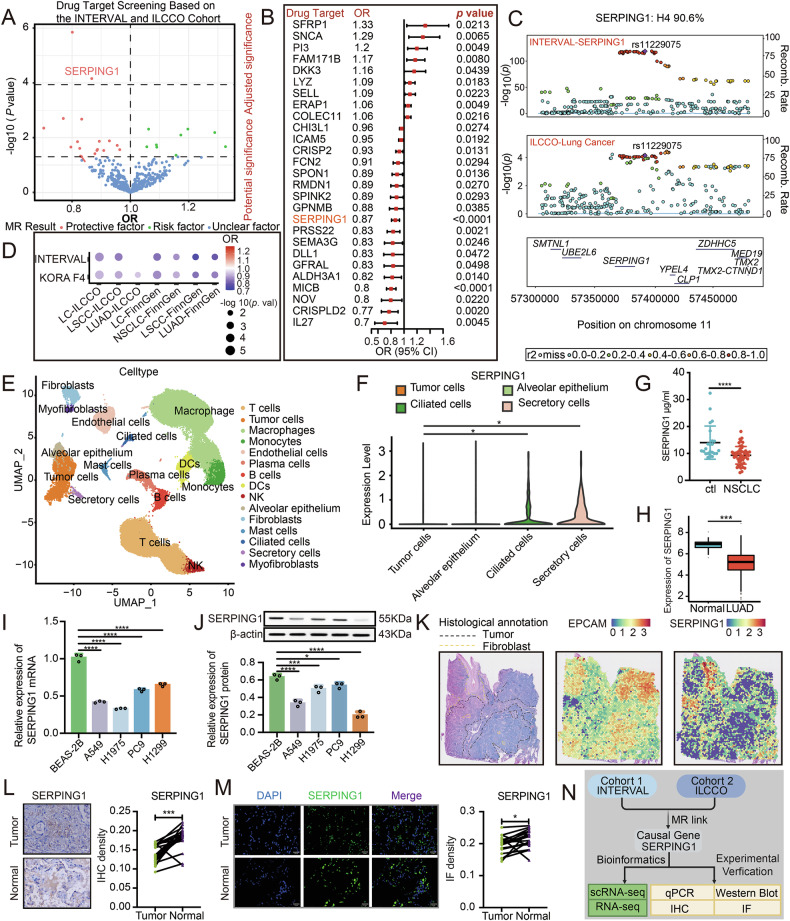
Integrated analysis detected that SERPING1 was lowly expressed in LUAD. **A** Volcano plots were used to visualize the causal effect of each circulating protein with the risk of developing LC. The upper line shows the adjusted significance while the bottom line refers to the potential significance. **B** Forest plot for displaying circulating proteins with statistically significant causal effect on the risk of LC. **C** Regional LocusZoom plots and colocalization analysis results for SERPING1. rs11229075 was used to proxy serum SERPING1 expression. **D** Using different cohorts to cross-validate the causal effect of the SERPING1 protein with the risk of LC. **E** UMAP visualization of identified cell clusters from tumor and normal samples by scRNA-seq. **F** Levels of SERPING1 in serval types of cells from scRNA-seq. **G** Quantification of SERPING1 in human plasma from healthy people (*n* = 23) and LUAD patients(*n* = 51) by ELISA assay. **H** Comparison of the expression of SERPING1 between cancer tissues and adjacent normal tissues from TCGA database. **I** qPCR and (**J**) western blot indicated the mRNA and protein levels of SERPING1 in cell lines, respectively. **K** Spatial images of HE with annotations (left) and spatial plots showed the spatial expression pattern of EPCAM (a tumor marker, middle) and SERPING1 (right). Expression of SERPING1 was detected by (**L**) IHC and (**M**) IF in LUAD tissues and adjacent normal tissues. **N** Workflow of these experiments.

In our quest to uncover circulating proteins associated with LC risk, we employed an MR analysis. Following the exclusion of proteins lacking genetic instrumentation from the LC GWAS dataset of the ILCCO cohort, our proteome-wide investigation involved 431 proteins. Notably, the *F* statistic for each genetic instrument surpassed 10 in the outcome data, indicating the absence of weak instrumental variable bias (Table S5). Post multiple testing correction, only two genetically predicted circulating proteins, namely SERPING1 and MHC class I polypeptide-related sequence B (MICB), emerged as significantly linked to LC risk (*p* < 0.05/431; Fig. 1A and Table S6). The odds ratio (OR) for LC per SD increase in genetically predicted levels stood at 0.87(95% confidence interval, CI: 0.81–0.93) (Fig. 1B). Additionally, strong genetic co-localization evidence was found for SERPING1 under both the prior and window (H4: SERPING1 90.6%; Fig. 1C). To validate these findings, we leveraged an additional proteomics cohort (KORA F4) and multiple LC cohorts (ILCCO and FinnGen). Our results consistently indicated that heightened SERPING1 expression correlated with reduced risk of LC and its subtypes across all cohorts (Fig. 1D and Table S7).

In delving deeper into the expression of SERPING1 in LUAD, we delved into scRNA-seq data to unveil its levels across various cell types (Fig. S1A, B). Through UMAP analysis based on cell-specific markers, we identified fifteen distinct cell types (Fig. 1E and Fig. S1C). Noteworthy differences were observed in SERPING1 levels, with tumor cells exhibiting lower expression compared to ciliated and secretory cells based on scRNA-seq (Fig. 1F). Moreover, the expression of SERPING1 in immune cells was shown in Fig. S1D. Notably, we detected the circulating protein levels of SERPING1 in human plasma from healthy people (*n* = 23) and NSCLC patients (*n* = 51), and found a lower expression in NSCLC patients, suggesting the low levels of SERPING1 were associated with NSCLC risk (Fig. 1G). Our analysis of raw mRNA matrix data extracted from TCGA database including adjacent normal tissues and LUAD tissues, and LUAD tissues revealed reduced expression levels in the LUAD tissues (Fig. 1H). Moreover, we consistently observed significantly higher mRNA (Fig. 1I) and protein levels (Fig. 1J) of SERPING1 in bronchial epithelial cells (BEAS-2B) compared to LUAD cells. Focusing on LUAD patients’ spatial transcriptomics data from SCAR, we utilized hematoxylin-eosin staining (HE) sections and the Epithelial cell adhesion molecule (EPCAM) cell marker to delineate fibroblast and tumor regions (Fig. 1K). Notably, we noted a decrease in SERPING1 levels within tumor regions. These findings were further validated through IHC (Fig. 1L) and IF (Fig. 1M) assays, underscoring the diminished SERPING1 levels in LUAD tumor tissues. The expression of SERPING1 across other cancer types was in Fig. S1E and levels of SERPING1 from culture supernatant were in Fig. S2. Collectively, our results signify the subdued expression of SERPING1 within LUAD (Fig. 1N).

### Low expression was linked to poor prognosis in LUAD patients and pathways associated with tumor proliferation

**Fig. 2:**
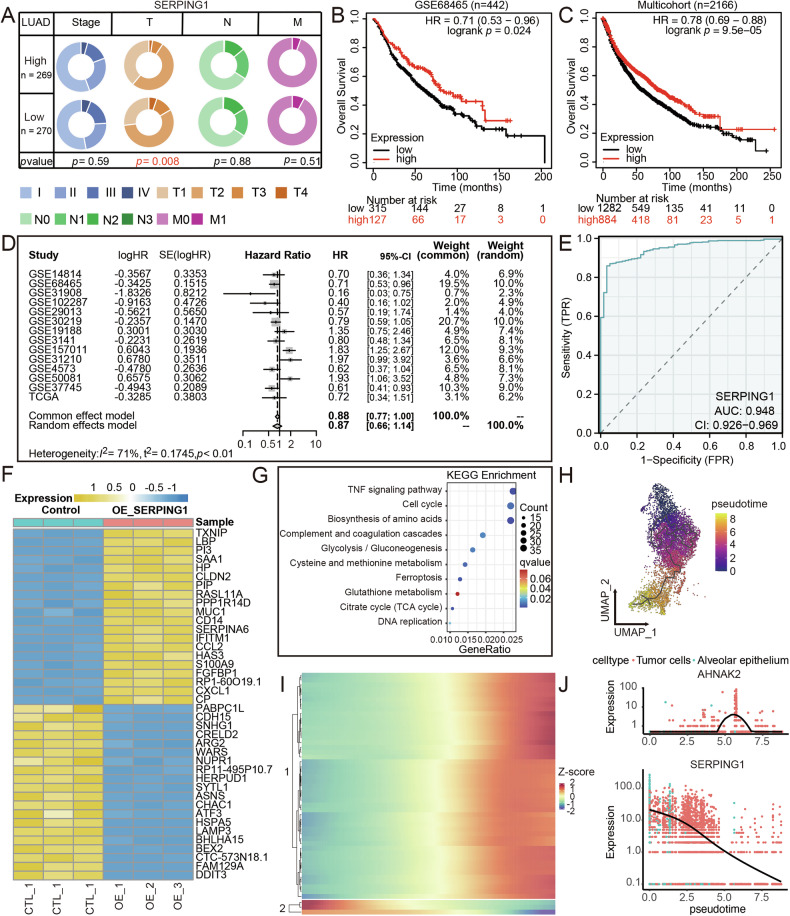
Low expression of SERPING1 was correlated with the poor prognosis of LUAD patients and proliferation pathways. **A** Comparison of distinct clinical features distribution in the SERPING1-high and low group from TCGA database. Kaplan-Meier curves of survival for SERPING1-high and low group from (**B**) GSE68465 (*n* = 442) and (**C**) multicohort study (*n* = 2166). **D** Forest plot of HR of SERPING1 expression and the prognosis of LUAD patients. **E** ROC curve of the predictive ability of SERPING1 expression level for LUAD diagnosis. **F** Heatmap of DEGs after overexpression of SERPING1 by RNA-seq. **G** KEGG analysis of DEGs; FDR < 0.05 was considered as significantly enriched. **H** Reconstructed developmental trajectory of tumor cells showing pseudotime colored from blue to yellow. **I** Heatmap showing expression of top 50 identified genes across single cells. The color key from blue to red indicates relative expression levels from low levels to high levels. **J** Expression patterns scatter plot of AHNAK2 and SERPING1 through pseudotime.

In our quest to unravel the impact of SERPING1 on the prognosis of LUAD patients, we observed a declining trend in SERPING1 levels corresponding to tumor size increments from T1 to T3 and T4 (Fig. 2A). Notably, within the LUAD cohort (Fig. 2B), individuals exhibiting elevated SERPING1 levels displayed a more favorable overall survival (OS) compared to those with diminished expression, a trend validated across multiple cohorts (Figs. 2C, D). Moreover, the diagnostic potential of SERPING1 in LUAD was outstanding (AUC = 0.948; Fig. 2E). Hence, the diminished expression of SERPING1 was correlated with an unfavorable prognosis for LUAD patients.

In the exploration of potential signaling pathways influenced by SERPING1, we conducted RNA-seq on A549 cells following infection with control and lentivirus to induce SERPING1 expression. DEGs subsequent to SERPING1 overexpression are depicted in Fig. 2F, with the enriched signaling pathways analyzed via KEGG. Results indicated a strong correlation between SERPING1 overexpression and tumor proliferation, encompassing pathways such as the tumor necrosis factor (TNF) signaling pathway, cell cycle and DNA replication (Fig. 2G). By analyzing values for individual cells, we derived a pseudotime trajectory that visually represented the developmental trajectory of tumor cells from scRNA-seq data, with colors transitioning from blue to yellow (Fig. 2H). A heatmap was used to exhibit the expression pattern of the top 50 dysregulated genes along this trajectory (Fig. 2I). Notably, the most dysregulated gene, AHNAK nucleoprotein 2 (AHNAK2), exhibited increased levels during tumor cell development in the control scenario, while SERPING1 expression decreased during the later stages of pseudotime (Fig. 2J). Therefore, SERPING1 demonstrated a negative correlation with proliferation in LUAD.

### SERPING1 suppressed LUAD progression in vitro

**Fig. 3:**
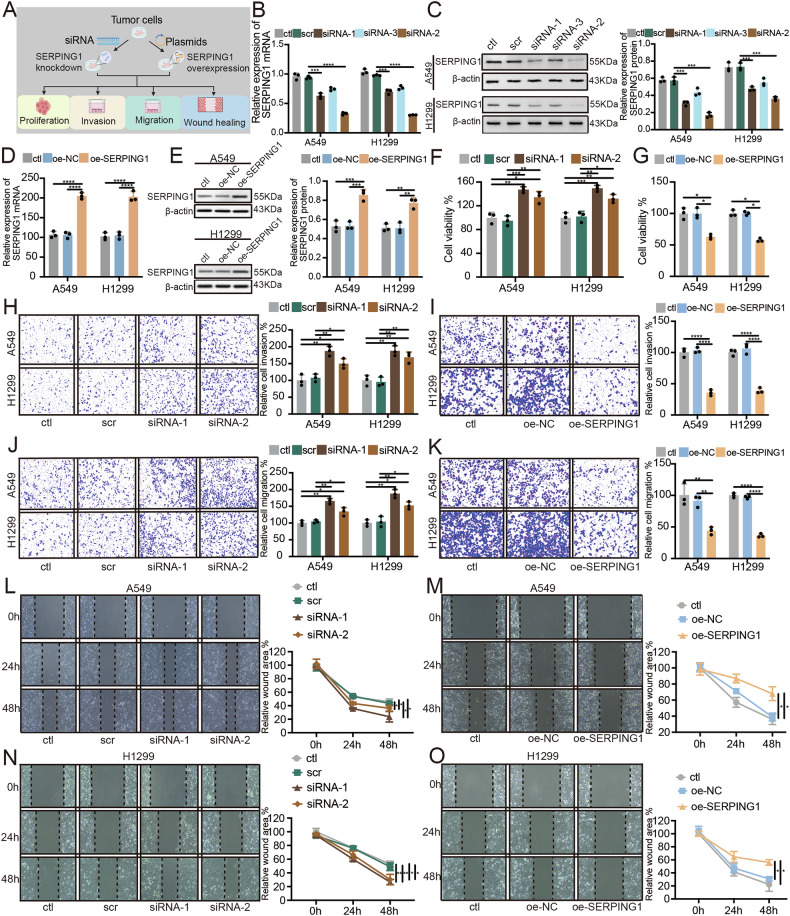
SERPING1 inhibited LUAD progression in vitro. **A** Workflow of this study. Levels of (**B**) mRNA and (**C**) protein of SERPING1 after knockdown via siRNA infection. Levels of (**D**) mRNA and (**E**) protein of SERPING1 after overexpression by plasmid transfection. Cell viability was measured by CCK-8 assays after SERPING1 (**F**) knockdown and (**G**) overexpression. Cell invasion was indicted by transwell assays after SERPING1 (**H**) knockdown and (**I**) overexpression. Cell migration was indicated by transwell assays after SERPING1 (**J**) knockdown and (**K**) overexpression. Wound healing assay results were shown in (**L**) A549 and (**N**) H1299 cells after SERPING1 knockdown and (**M**) A549 and (**O**) H1299 cells after SERPING1 overexpression. In each panel, representative images and quantification graphs were shown.

To demonstrate the biological functions of SERPING1 in LUAD, we conducted investigations into the impact of SERPING1 knockdown and overexpression on the proliferation, migration, invasion, and wound healing of LUAD cells (Fig. 3A). SERPING1 levels were knocked down through siRNA infection (Figs. 3B, C), and elevated via plasmid transfection (Figs. 3D, E). Post SERPING1 knockdown, LUAD cell proliferation was augmented (Fig. 3F), whereas proliferation was hindered following SERPING1 overexpression (Fig. 3G). Similarly, cell invasion was heightened after SERPING1 knockdown (Fig. 3H) and curtailed after SERPING1 overexpression (Fig. 3I). Transwell migration assays also revealed an increase in cell migration post SERPING1 knockdown (Fig. 3J) and a decrease after SERPING1 overexpression (Fig. 3K). Wound healing assays demonstrated that SERPING1 knockdown promoted the migration of A549 (Fig. 3L) and H1299 (Fig. 3N) cells, while SERPING1 overexpression attenuated the migration of A549 (Fig. 3M) and H1299 (Fig. 3O) cells. These findings collectively suggest that SERPING1 functions as a tumor suppressor gene, impeding the progression of LUAD in vitro.

### SERPING1 suppressed LUAD progression in vivo

**Fig. 4:**
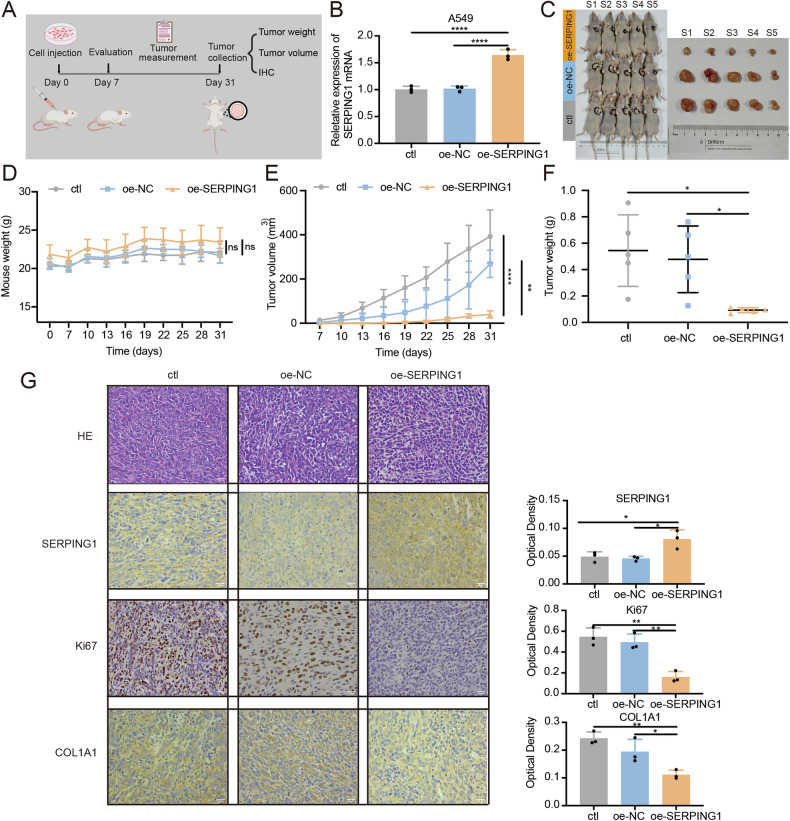
SERPING1 restrained LUAD progression in vivo. **A** Schematic of anticancer effect of SERPING1 in vivo. **B** Lentiviruses were infected into A549 cells to stably overexpress SERPING1. **C** Representative images of mice-bearing tumors derived from xenograft nude mouse model. **D** Weight of mouse in three groups. **E** Tumor volume and (**F**) weight in the three groups were measured every 3 days. **G** HE stained and IHC detection of serial sections from Nude mouse xenograft model. NC, negative control; oe-NC, an empty-lentivirus vector; oe-SERPING1, overexpression of SERPING1.

To validate the tumor-suppressing effects of SERPING1 in vivo, we executed a nude mouse xenograft model (Fig. 4A). A549 cells were infected with lentiviruses to establish stable SERPING1 overexpression (Fig. 4B). Subsequently, nude mice were subcutaneously injected with A549 cells to establish a tumor-bearing model. Tumor volume and body weight measurements were conducted every 3 days, with mice being sacrificed after 31 days post-inoculation (Fig. 4C). Notably, no statistical difference was found in the mouse body weight across the various groups (Fig. 4D). Remarkably, tumors exhibited significantly reduced size (Fig. 4E) and weight (Fig. 4F) after SERPING1 overexpression compared to controls. Additionally, high expression of SERPING1 and low expression of Ki67 and Collagen I (COL1A1) was investigated by IHC after SERPING1 overexpression in vivo (Fig. 4G). These results underscore the inhibitory role of SERPING1 in LUAD progression in vivo.

### SERPING1 upregulated TSC2 expression to inhibit the mTOR/4E-BP1 axis

**Fig. 5:**
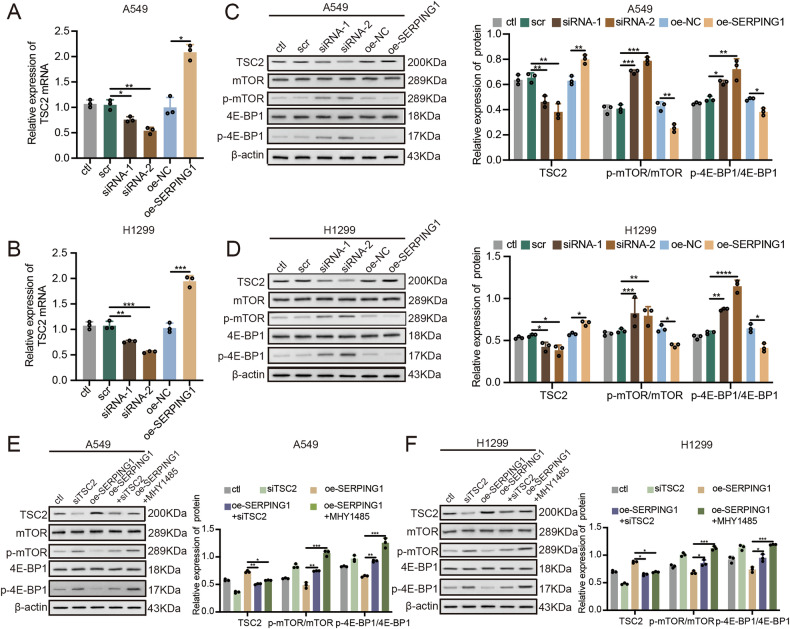
SERPING1 upregulated the levels of TSC2 expression to suppress mTOR/4E-BP1 axis. Levels of TSC2 mRNA were measured by qPCR in (**A**) A549 and (**B**) H1299 cells after SERPING1 knockdown and overexpression. Protein levels of TSC2, mTOR, p-mTOR, 4E-BP1, and p-4E-BP1 in (**C**) A549 and (**D**) H1299 cells after SERPING1 knockdown and overexpression. Protein levels of TSC2, mTOR, p-mTOR, 4E-BP1, and p-4E-BP1 in (**E**) A549 and (**F**) H1299 cells after TSC2 knockdown and MHY1485 (mTOR activator) treatment.

In our pursuit to unravel the potential mechanism underpinning the impact of SERPING1 in LUAD, we validated the alterations in downstream gene expression following RNA-seq analysis (Fig. 2F). Notably, Tuberous Sclerosis Complex 2 (TSC2) exhibited upregulation following SERPING overexpression as indicated by the RNA-seq results. In A549 cells, mRNA levels of TSC2 were diminished post-SERPING1 knockdown and amplified post-SERPING1 overexpression (Fig. 5A), which was found in H1299 cells as well (Fig. 5B). Remarkably, phosphorylated levels of p-mTOR and p-4E-BP1 were heightened subsequent to SERPING1 knockdown (Fig. 5C). Conversely, TSC2 expression was augmented, while p-mTOR and p-4E-BP1 levels were decreased following SERPING1 overexpression (Fig. 5C). The consistent results were mirrored in H1299 cells (Fig. 5D). Meanwhile, we conducted rescue experiments by knocking down TSC2 with SERPING1 overexpression in LUAD cells. In A549 cells, knockdown of TSC2 led to the increase of p-mTOR and p-4E-BP1 after SERPING1 overexpression (Fig. 5E), which was similar in H1299 cells (Fig. 5F). Subsequently, we employed the mTOR activator, MHY1485, as a positive control to treat cells. Remarkably, we observed that MHY1485 reversed the SERPING1-induced decrease in p-mTOR and p-4E-BP1 expression in both LUAD cell lines (Fig. 5E, F), aligning with the outcomes of TSC2 knockdown. We found the consistent results in the mTOR/4E-BP1 pathway after the treatment of recombinant SERPING1 and supernatant from SERPING1-overexpressed cells (Fig. S3). Meanwhile, MHY1485 reversed the inhibition of SERPING1 overexpression on the progression of LUAD cells (Fig. S4). In conclusion, our findings suggest that SERPING1 enhances TSC2 levels to suppress the mTOR/4E-BP1 axis, shedding light on a potential regulatory mechanism in LUAD progression.

### SERPING1-mediated inhibition of LUAD progression via TSC2 upregulation

**Fig. 6:**
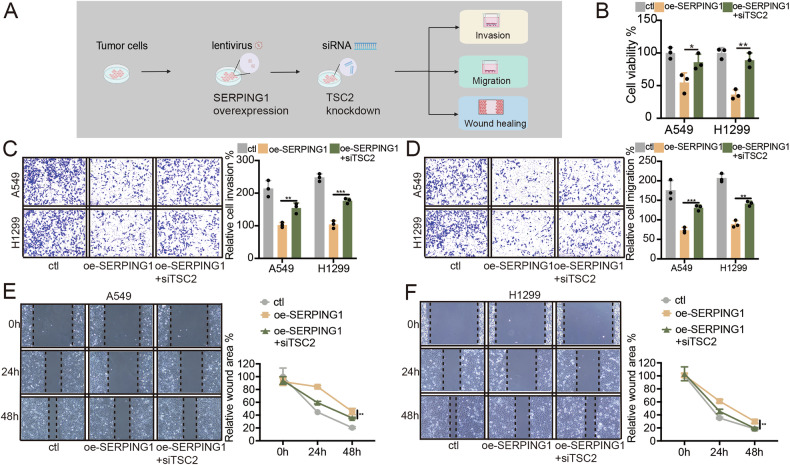
SERPING1 inhibited invasion and migration by inhibiting TSC2/mTOR/4E-BP1 axis. **A** Workflow of this study. **B** Cell viability was measured by CCK-8 assays after SERPING1 overexpression and TSC2 knockdown. **C** Cell invasion and **D** migration were indicted by transwell assays after SERPING1 overexpression and TSC2 knockdown. Wound healing assay results were shown in (**E**) A549 and (**F**) H1299 cells after SERPING1 overexpression and TSC2 knockdown.

In our investigation, we explored how SERPING1 suppresses the progression of LUAD by modulating TSC2. To assess whether the progression induced by SERPING1 was linked to reduced TSC2 expression, we conducted TSC2 knockdown in LUAD cells with SERPING1 overexpression (Fig. 6A). TSC2 knockdown reversed cell viability after SERPING1 overexpression (Fig. 6B). The results showed that TSC2 knockdown significantly enhanced invasion, a phenomenon counteracted by SERPING1 overexpression (Fig. 6C). Additionally, both transwell migration assays (Fig. 6D) and wound healing assays (Fig. 6E, F) demonstrated that TSC2 knockdown reversed the migratory effects induced by SERPING1 overexpression in both LUAD cell lines. Hence, our findings suggest that SERPING1 inhibits LUAD progression by regulating TSC2.

### Facilitation of SERPING1 transcription by transcription factor SP5 binding to the SERPING1 gene promoter

**Fig. 7:**
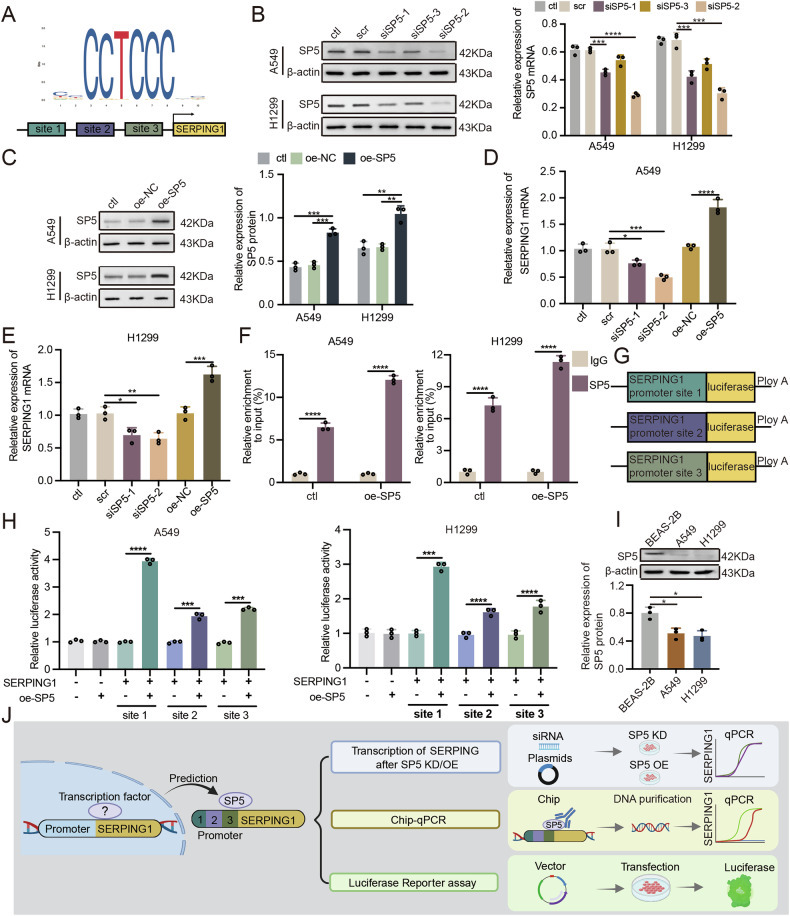
SERPING1 transcription was stimulated by SP5 direct binding to the SERPING1 gene promoter. **A** Potential sites binding with SP5 were predicted in the promoter of SERPING1 from JASPAR. **B** SiRNAs were infected into A549 and H1299 cells to knock down SERPING1. **C** Plasmids were transfected into A549 and H1299 cells to overexpress SP5. Levels of SERPING1 mRNA after SP5 knockdown and overexpression in (**D**) A549 and (**E**) H1299 cells. **F** CHIP-qPCR revealed the enrichment of the SERPING1 promoter without or with SP5 overexpression. The promoter of SERPING1 includes the site 1-3. **G** Plasmids containing three sites from the SERPING1 promoter were used for luciferase reporter assays. **H** Luciferase reporter assays detected the luciferase activity of three sites from the SERPING1 promoter after SP5 overexpression. **I** Western blotting indicated SP5 protein levels in BEAS-2B and LUAD cells. **J** Schematic diagram of exploring the SERPING1 gene upstream regulator.

In our quest to identify key regulators of SERPING1 transcription, we utilized JASPAR software to predict potential transcription factors of SERPING1. Specificity Protein 5 (SP5) emerged as the top candidate, with JASPAR software predicting three SERPING1-binding sites in the promoter region (Fig. 7A). Subsequently, we employed siRNAs to knockdown SP5 (Fig. 7B) and transfected plasmids into LUAD cells to overexpress SERPING1 (Fig. 7C). Decreased SERPING1 transcription following SP5 knockdown and increased transcription post SP5 overexpression were observed in both A549 and H1299 cells (Fig. 7D, E). Furthermore, our CHIP-qPCR analysis revealed a significant enrichment of SP5 at the SERPING1 promoter region. Noteworthy, overexpression of SP5 facilitated the enrichment of the SERPING1 promoter sequence, indicating specific binding of SP5 to the SERPING1 promoter including the site 1-3 (Fig. 7F). Luciferase reporter assays were then conducted to confirm the binding activity of SP5 to the SERPING1 promoter, with plasmids containing potential binding sites from the SERPING1 promoter (site 1, 2, 3) being utilized (Fig. 7G). Overexpression of SP5 in LUAD cells significantly increased luciferase activities, highlighting the importance of these three sites for SP5’s specific binding to the SERPING1 promoter (Fig. 7H). Our analysis revealed higher expression levels of SP5 in BEAS-2B cells compared to LUAD cells, aligning with the expression pattern of SERPING1 (Fig. 7I). Therefore, these results suggest that the transcription of the SERPING1 gene is activated through the direct binding of SP5 to the SERPING1 promoter (Fig. 7J).

### Predicting immunotherapeutic benefits for cancer patients through SERPING1 expression

**Fig. 8:**
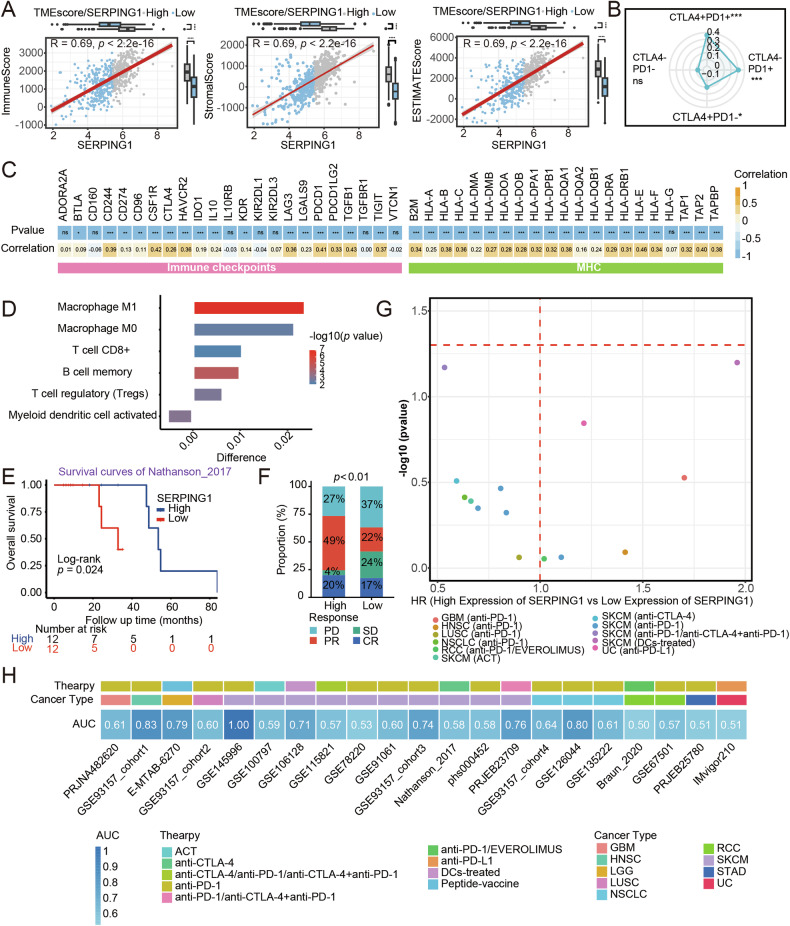
Patients with SERPING1-high levels exhibited remarkable benefits from immunotherapy. **A** Correlation between immune score, stromal score, ESTIMATE score, and SERPING1 in the high and low SERPING1 group. The expression of SERPING1 in the high- and low-score groups was on the top, while the scores in the SERPING1-high and -low groups were on the right. **B** Satellite maps were used to represent the correlation between the four IPS scores and the expression of SERPING1. **C** Correlation analysis for SERPING1 and the expression of MHC family genes and immune checkpoints. **D** Difference in the relative abundance of immune cell infiltration in tumor microenvironment between the SERPING1-high and -low groups. Difference > 0 indicates that the immune cells were enriched in the SERPING1- high group and the column color represents the statistical significance of the difference. **E** Prognosis of the patients receiving immunotherapy (anti-CTLA-4) in the cohort of Nathanson (2017). **F** Proportion of patients responding to anti-PD-1 therapy in the SERPING1-high and low group from the cohort of PRJE23709. **G** Analysis of survival differences between SERPING1-high and low expression in different immunotherapy cohorts. HR < 1 in the volcano plot indicates that patients with SERPING1-high expression have a better prognosis than patients with SERPING1-low expression. **H** Predictive power of SERPING1 expression levels for efficacy in different immunotherapy cohorts. A larger value in the heatmap indicates greater predictive ability. CR, complete response; PD, progressive disease; PR, partial response; SD, stable disease.

In our quest to uncover the potential roles of SERPING1 in immunotherapy, we delved into the association between SERPING1 expression and immune-related scores. Notable, Immune, stromal, and ESTIMATE scores were significantly elevated in the SERPING1-high group (Fig. 8A). Furthermore, the levels of SERPING1 displayed a remarkable positive correlation with IPS scores, particularly in the context of immunotherapy involving anti-CTLA-4 and anti-PD-1 treatments (Fig. 8B). Moreover, the expression of SERPING1 exhibited a robust correlation with immune checkpoints and MHC (Fig. 8C). Additionally, SERPING1 levels were found to be linked with the expression of immune receptors (Fig. S5A) and chemokines (Fig. S5B). We also explored the differences in TME cells between the SERPING1-high and low groups. Our analysis revealed that the SERPING1-high group exhibited increased infiltration levels of M1 and M0 macrophages, CD8 + T cells, memory B cells, and regulatory T cells, while the SERPING1-low group showed elevated levels of myeloid dendritic cells (Fig. 8D). Similarly, in the cohort study of Nathanson (2017), patients with high SERPING1 levels demonstrated a more favorable prognosis (Fig. 8E). Additionally, the proportion of patients responding to immunotherapy was notably higher in the SERPING1-high group compared to the SERPING1-low group (Fig. 8F). Across various cohorts receiving immunotherapy, the SERPING1-high group tended to experience superior survival benefit when contrasted with the SERPING1-low group (Fig. 8G). Interestingly, an intriguing finding emerged when analyzing the values of AUC across immunotherapy-focused cohorts, which ranged from 0.5 to 1. This range suggests that SERPING1 serves as a predictive biomarker for immunotherapy response (Fig. 8H).

### Safety assessment: SERPING1 overexpression and cardiometabolic risks

In light of our prior discoveries indicating that SERPING1 overexpression could serve as a novel approach to impede tumor growth and extend survival, the evaluation of potential side effects and alternative indications of SERPING1 overexpression became paramount before contemplating clinical implementation. Notably, minimal adverse events, such as elevated body temperature, were reported [24, 25]. To delve deeper, we investigated the causal relationship between genetically predicted SERPING1 overexpression and 17 cardiometabolic traits alongside 11 cardiovascular diseases.

Our analysis unveiled a significant association (*p* < 0.0018) between SERPING1 overexpression and elevated systolic blood pressure (OR = 1.41) and glycosylated hemoglobin (HbA1c; OR = 1.01), while a weak correlation was observed with decreased body mass index (BMI; OR = 0.99, *p* = 0.01; Fig. S3A). Conversely, the genetic proxy for SERPING1 overexpression did not exhibit statistically significant links with other lipids, blood pressure, blood glucose, and anthropometric measures (Fig. S6A). Furthermore, aside from a modest correlation with the risk of developing type I diabetes (OR = 1.13, *p* = 0.03), the association between genetically predicted SERPING1 overexpression and the remaining cardiovascular diseases did not reach statistical significance (Fig. S6B). In summary, our findings suggest a reassuring safety profile when considering the application of SERPING1 overexpression for the future treatment of LC, with no substantial increase in cardiometabolic risks or susceptibility to cardiovascular diseases identified in our comprehensive assessment.

## Discussion

Our investigation revealed a significant correlation between elevated levels of the circulating protein SERPING1 and a remarkable reduction in LC risk, as evidenced by the analysis of extensive GWAS data. Furthermore, we conducted a thorough examination of SERPING1 expression levels in LC, assessing its prognostic, diagnostic, and immunotherapeutic predictive values. Additionally, both in vitro and in vivo experiments were undertaken to validate the tumor-suppressing capabilities of SERPING1 specifically and regulation of TSC2/mTOR pathway in LUAD. Finally, we delved into the safety considerations surrounding SERPING1 overexpression through MR analysis, laying the groundwork for potential clinical applications targeting SERPING1.

In our quest to identify potential targets for LC treatment, we recognized the substantial costs associated with surveying numerous proteins in large-scale preclinical sample studies. To address this challenge, our methodology provided a strategic solution by prioritizing candidate biomarkers using data from large-scale GWAS analyses. Noteworthy are MR studies, focusing on circulating biomarkers, which often validate or predict outcomes observed in extensive drug intervention trials that impact biomarker levels [26, 27]. Our MR analysis in this study highlighted that heightened SERPING1 expression was associated with a decreased LC risk. Moreover, MR analyses were also conducted to pinpoint therapeutic targets for various conditions such as aortic aneurysms [11] and breast cancer [28], showcasing the potential of this approach as a novel and effective strategy for screening pharmaceutical targets across different diseases.

SERPING1, is a protease inhibitor categorized within the serine superfamily, functions to deactivate the C1r and C1s proteases housed within the C1 complex of the complement classical pathway [29, 30]. While SERPING1 mutations have been extensively linked to type I and II Hereditary angioedema (HAE), its implications in oncology have remained relatively unexplored [31–34]. Our investigation revealed an upregulation of both mRNA and protein levels of SERPING1 in normal lung tissues in contrast to LUAD tissues. Intriguingly, diminished SERPING1 expression correlated with advanced T-stage and poor survival outcomes in LUAD patients. This trend mirrored findings in prostate cancer and breast cancer, where reduced SERPING1 expression corresponded to higher pathological grades, advanced tumor stages [16], and unfavorable prognoses [35]. Noteworthy is our discovery of SERPING1’s potential in early bone metastasis detection in breast cancer, aligning with its diagnostic significance in LUAD [35]. A plausible regulatory mechanism we identified suggests that decreased SERPING1 expression in tumor cells might hyperactivate the classical complement pathway, thereby promoting lung carcinogenesis and progression. To validate our bioinformatics insights, we conducted comprehensive in vitro and in vivo experiments, unveiling strong correlations between SERPING1 and cell proliferation, metabolic processes, and immune responses, hinting at its diverse biological functions warranting further exploration.

In our quest to delve into the molecular mechanism of SERPING1 in LUAD, we conducted RNA-seq to uncover downstream regulators. Our investigation unveiled that SERPING1 acts as a positive regulator of TSC2, a pivotal player in the mTOR singling pathway. Through validation experiments, we confirmed that SERPING1 exerts a suppressive effect on LUAD progression by modulating the mTOR pathway via TSC2. Consistent with prior research [36–38], which links mTOR pathway inhibition to reduced tumor proliferation, invasion and migration, our findings underscore the therapeutic potential of targeting this pathway in LUAD. Furthermore, our exploration extended to the upstream regulators of SERPING1, revealing a novel link with SP5. Our research demonstrated that SP5 directly interacts with the SERPING1 promoter, influencing SERPING1 transcription and thereby contributing to the different expression of SERPING1 in normal lung tissues and LUAD tissues. Notably, SP5’s association with the activation of the AKT/mTOR signal pathway in prostate cancer [39], hints at intriguing regulatory mechanisms governing SERPING1 transcription that warrant further investigation. Interestingly, SP5 is found to be the target of the WNT pathway [40] and negatively regulates WNT transcriptional programs via repressing the expression of Wnt3a [41]. In combination with our findings, SP5 was identified as the novel linking element between mTOR and WNT/β-catenin signaling pathway, suggesting the potential of multi-pathway targeted therapies against LC.

Transitioning towards potential treatment avenues and clinical applications of SERPING1 in LUAD, we assessed immunotherapy responses based on the expression of SERPING1. Our results indicated that patients with low SERPING1 expression exhibited shorter survival times, suggesting a potential stratification strategy for identifying LUAD patients who may benefit from immunotherapy. Drawing parallels with the use of Serpin Family H member 1 (SERPINH1) in developing an immunotherapy efficacy scoring system for osteosarcoma patients [42], the influence of SERPING1 on immunotherapy outcomes could be attributed to its role in complement system activation, pivotal in immune surveillance against malignant cells. Our findings suggest that combining SERPING1 overexpression with immune checkpoint inhibitors could potentiate therapeutic outcomes for LUAD patients. Notably, immune checkpoint inhibitors stimulate cytotoxic T cell proliferation, while SERPING1 overexpression diminishes Myeloid-Derived Suppressor Cells (MDSCs) infiltration in the tumor microenvironment, alleviates MDSC-induced T cell suppression, and enhances overall T cell function. These insights underscore the potential of SERPING1 as a promising target for LC therapy, offering new avenues for personalized treatment strategies in the fight against this malignancy.

In the realm of therapy for HAE, both intravenous and subcutaneous administrations of plasma-derived SERPING1 have proven effective as complement inhibitors [43, 44]. However, the known occurrence of local reactions at injection site poses a common challenge with these treatments. When considering their application in tumor treatment, particularly for LC patients, heightened attention is warranted due to potential cardiovascular events that could disrupt the delivery of essential therapies like radiotherapy and immunotherapy. Our observations revealed that while genetically predicted SERPING1 overexpression correlated with elevated systolic blood pressure and glycosylated hemoglobin levels, no definitive links to other cardiovascular risks were apparent. These initial findings underscore the imperative for further exploration in this domain.

Several limitations inherent in our study necessitate careful consideration when interpreting our results. Firstly, the ethnic homogeneity of our study cohort highlights the need for broader investigations to validate the generalizability of our findings across diverse ancestries beyond European descent. Secondly, exploring reverse causation was impeded by the lack of SNP mapping between the IVs associated with LC and circulating SERPING1 proteins. Despite this, the inherent nature of MR offers some protection against reverse causation biases. Thirdly, the potential for a non-linear effect of SERPING1 on LC risk cannot be discounted. Given the likelihood that SERPING1 might exert varying effects at different expression levels, future clinical trials are essential to determine the optimal therapeutic efficacy and safety profile. Lastly, elucidating the precise mechanism through which SERPING1 overexpression modulates LC risk mitigation warrants comprehensive examination and discussion in future research endeavors.

In conclusion, our investigation unveiled that genetically predicted heightened levels of circulating SERPING1 correlated with a reduced risk of LC. Additionally, we delved into the potential of SERPING1 as a tumor suppressor gene, exploring its diagnostic, prognostic, and therapeutic implications in LC through multi-omics analyses and fundamental experiments. In summary, these discoveries shed light on SERPING1 as a promising novel target for LC. Moving forward, randomized trials will be imperative to assess the efficacy and safety of targeting SERPING1 for the prevention and treatment of LC (Fig. S6C).

## Supplementary information

Supplementary figures

Supplementary tables

Full unedited gel

## Data Availability

The GWAS summary data used in this study are publicly available via the IEU OpenGWAS database (https://gwas.mrcieu.ac.uk/). In addition, multi-omics data are available by accessing the TCGA database (https://portal.gdc.cancer.gov/) and the GEO database (https://www.ncbi.nlm.nih.gov/).
