# Supplementary material for: WNT inhibitor SP5-mediated SERPING1 suppresses lung adenocarcinoma progression via TSC2/mTOR pathway
Source: Cell Death Dis. 2025 Feb 17;16(1):103. doi: 10.1038/s41419-025-07440-3 (PMC11832940; doi:10.1038/s41419-025-07440-3)
Supplement: Supplementary file 1 — Supplementary figures [file 41419_2025_7440_MOESM1_ESM.pdf]

WNT inhibitor SP5-mediated SERPING1 suppresses lung adenocarcinoma progression via TSC2/mTOR pathway

Running title: tumor suppressor gene SERPING1 in LUAD

Yefeng Shen[1,2,#], Xiaofeng Dong[3,#], Xujia Li[4], Zhiyuan Shi[5], Tingting Shao[6], Junlan Jiang[7,8], Jian Song[1,9,*]

[1]Institute of Cardiovascular Sciences, Guangxi Academy of Medical Sciences, Nanning, China; [2]Department of Thoracic Surgery, Beijing Friendship Hospital, Capital Medical University, Beijing, China; [3]Department of Hepatobiliary, Pancreas and Spleen Surgery, the People's Hospital of Guangxi Zhuang Autonomous Region (Guangxi Academy of Medical Sciences)，Nanning，China; [4]State Key Laboratory of Oncology in South China, Guangdong Provincial Clinical Research Center for Cancer, Sun Yat-sen University Cancer Center, Guangzhou, China; [5]School of Pharmaceutical Science and Technology, Faculty of Medicine, Tianjin University, Tianjin, China; [6]Department of Pediatrics, Peking University First Hospital, Beijing, China; [7]Department of Pathology, the first Affiliated Hospital, Anhui Medical University, Hefei, China; [8]Pathology Center, Anhui Medical University, Hefei, China; [9]Department of Radiation Oncology, Renji Hospital, School of Medicine, Shanghai Jiao Tong University, Shanghai, China.

*Correspondence author: Jian Song, Institute of Cardiovascular Sciences, Guangxi Academy of Medical Sciences, 6 Taoyuan Road, Nanning, Guangxi Zhuang autonomous region, 530021, China. Email: jsong@gxams.org.cn.

#Co-first author.

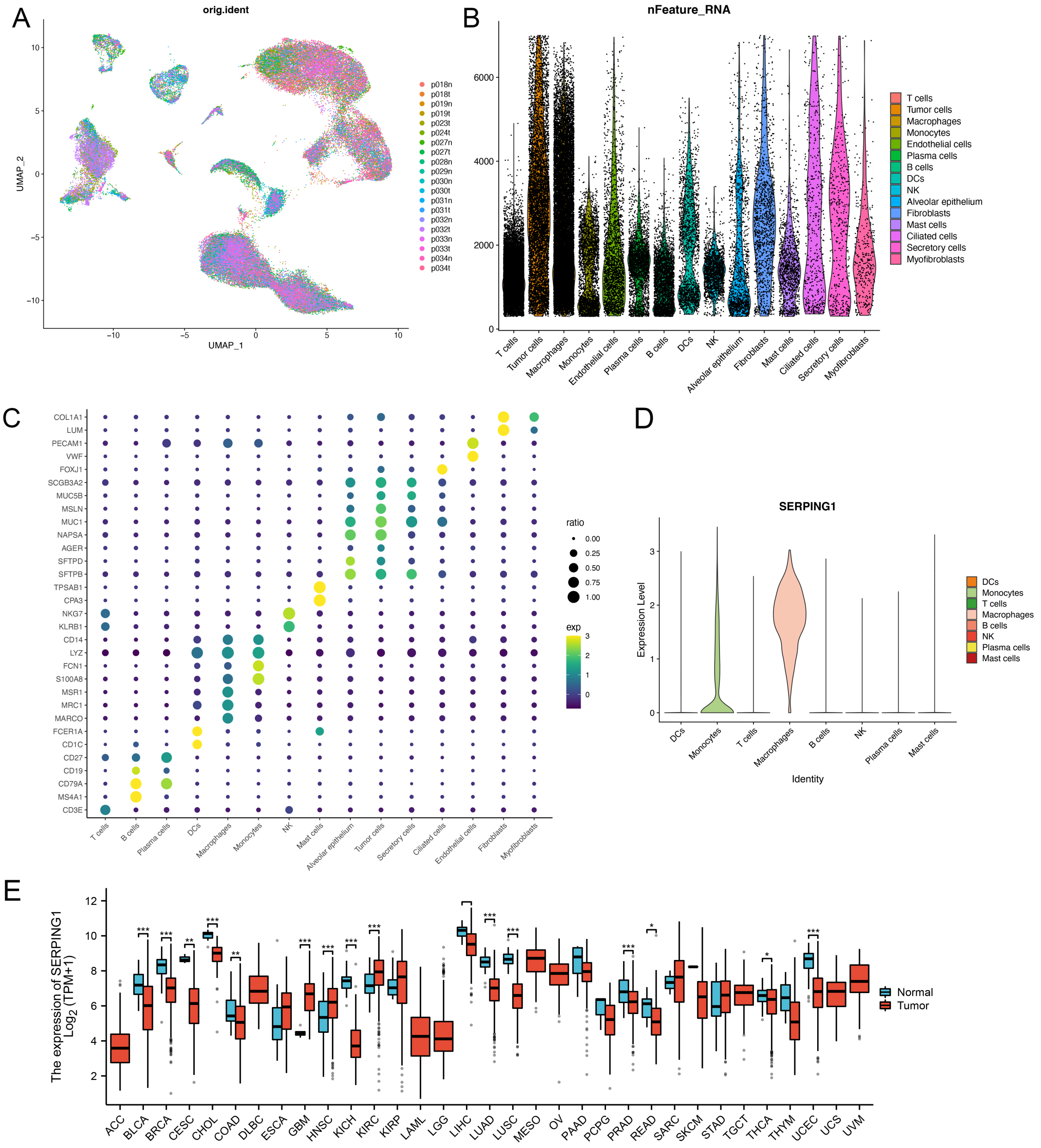

Fig. S1. Identification of cell clusters from scRNA-seq. (A) UMAP shows the distribution of cells from samples. (B) Post-quality control filtering of each sequenced cell, which was plotted in violin plots to display their number of RNA features (nFeature_RNA). (C) Bubble plot of cell markers of each cell cluster. Dot size represented abundance while the color represented the expression levels. (D)

Expression of SERPING1 in immune cells from scRNA-seq. (E) Levels of SERPING1 in 33 types of cancer and adjacent tissues.

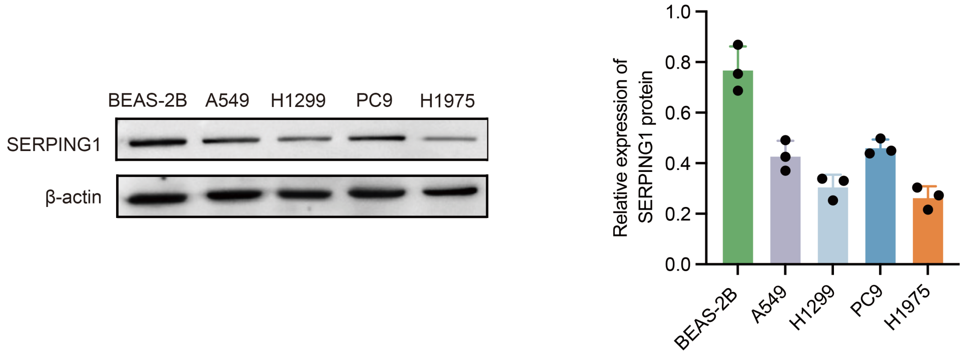

Fig. S2. Western blot detection of SERPING1 from the supernatant of indicated cells.

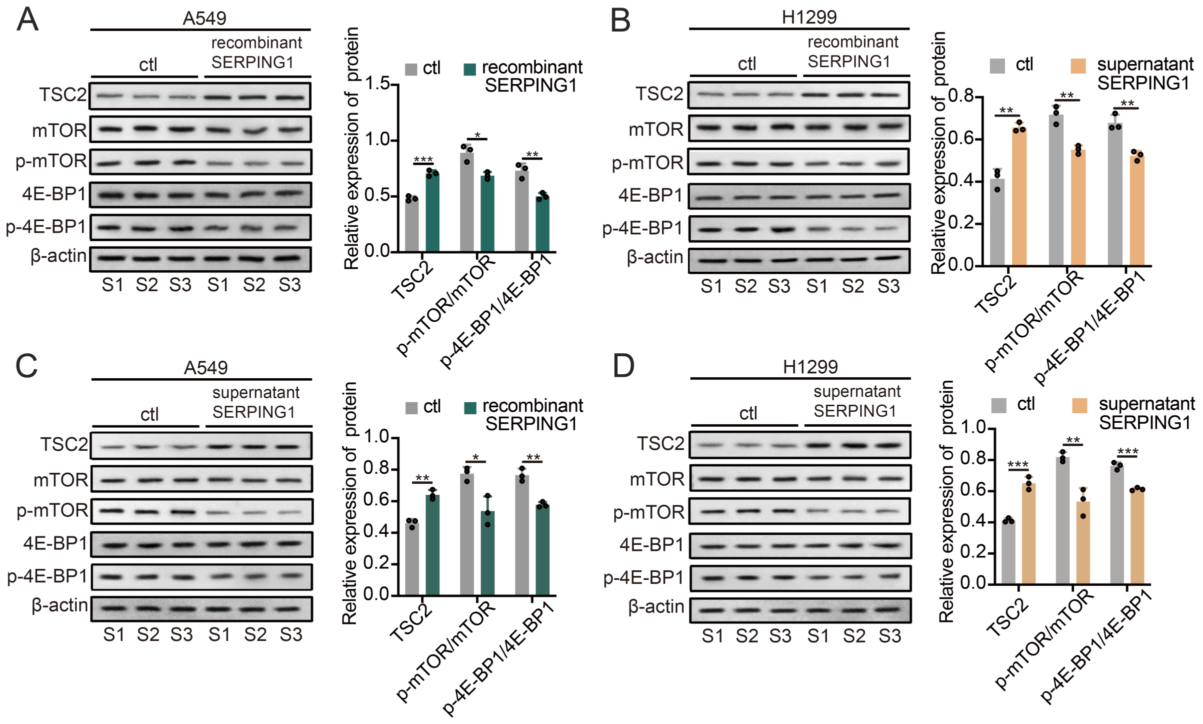

Fig. S3. Regulation of mTOR pathway after the treatment of recombinant SERPING1 and supernatant from SERPING1-overexpressed cells. Protein levels of TSC2, mTOR, p-mTOR, 4E-BP1, and p-4E-BP1 after the treatment of (A, B) recombinant and (C, D) supernatant SERPING1. The triplicate of samples is shown in S1, S2 and S3.

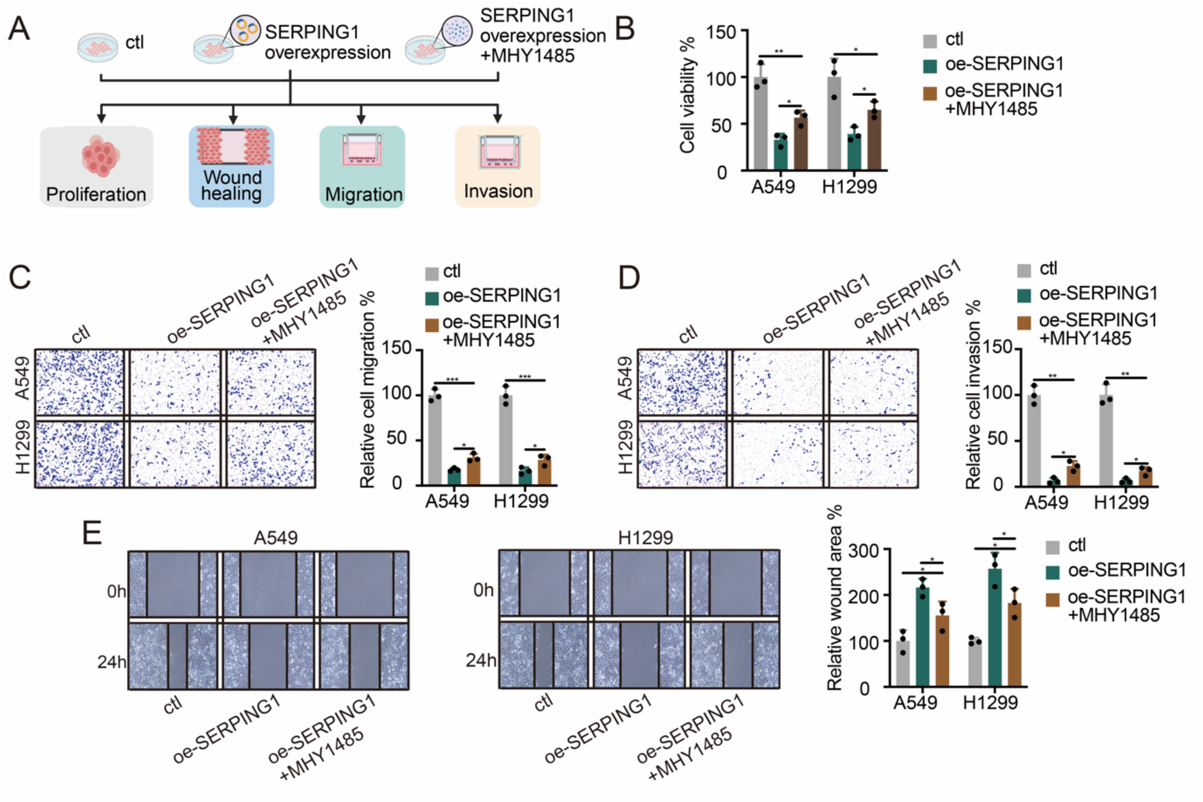

Fig. S4. MHY1485 reversed the inhibition of SERPING1 overexpression on the progression of LUAD cells. (A) Workflow of this study. (B) CCK8 assay indicated the cell viability of cells after SERPING1 overexpression and MHY1485 treatment. (C) The migration, (D) invasion and (E) wound healing of LUAD cells after SERPING1 overexpression and MHY1485 treatment.

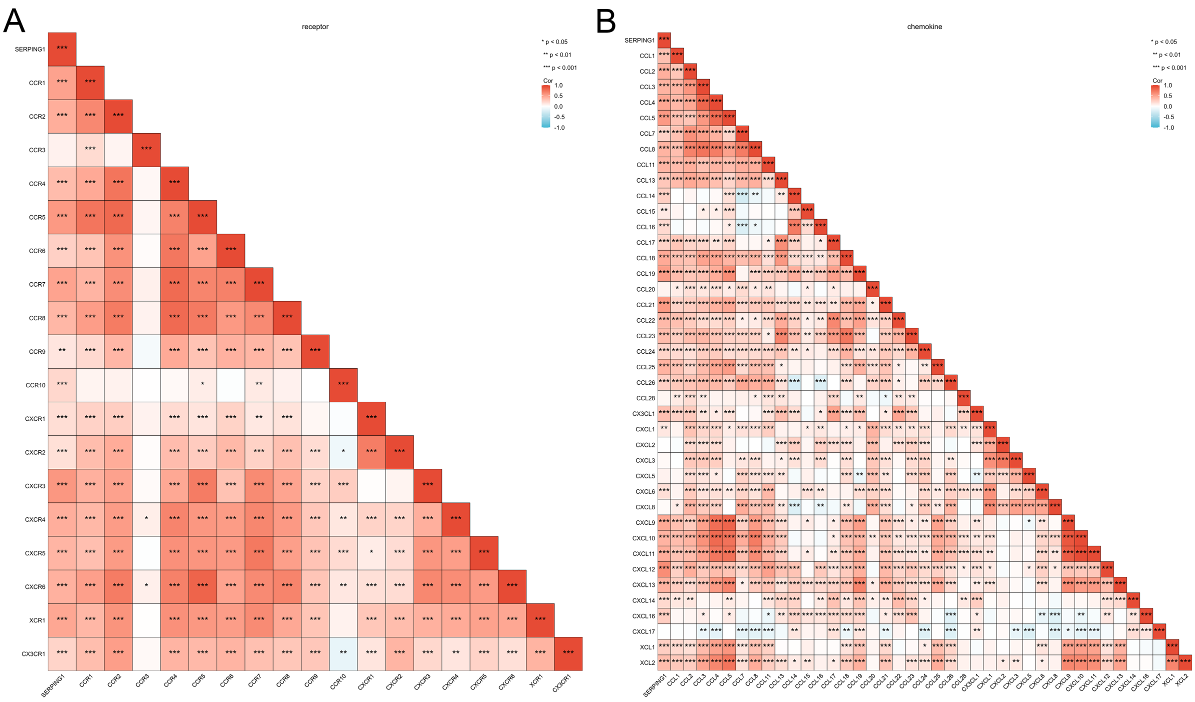

Fig. S5. Correlations between SERPING1 and (A) immune receptors and (B) chemokines.

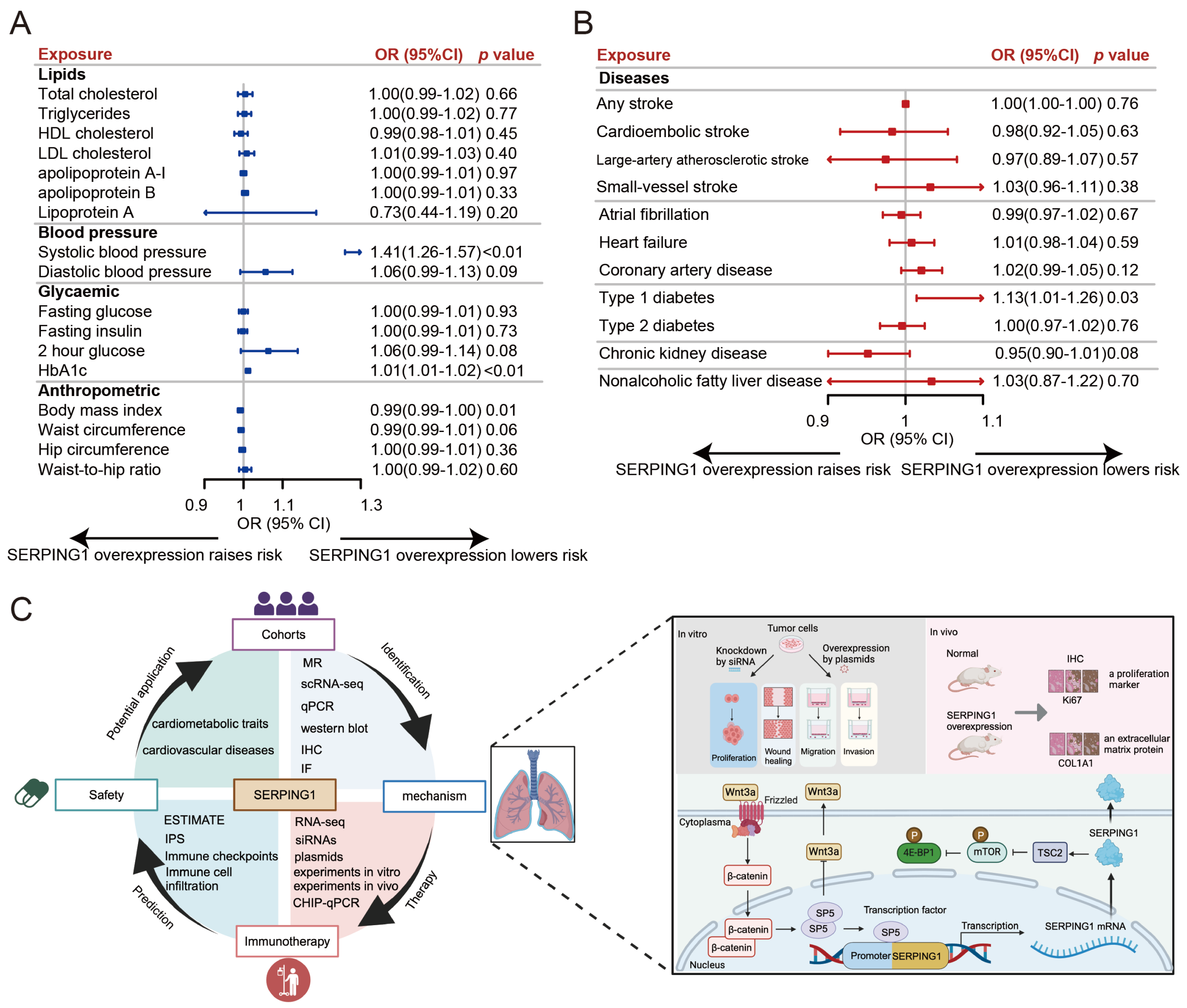

Fig. S6. Correlation between genetically predicted SERPING1 and other cardiovascular conditions. The forest plot shows MR effect estimates and 95% IC for the genetic proxied antagonistic effect of SERPING1 and (A) 17 cardiometabolic traits and (B) 11 cardiovascular diseases. (C) Schematic diagram of this study.
