# Supplementary material for: WNT inhibitor SP5-mediated SERPING1 suppresses lung adenocarcinoma progression via TSC2/mTOR pathway
Source: Cell Death Dis. 2025 Feb 17;16(1):103. doi: 10.1038/s41419-025-07440-3 (PMC11832940; doi:10.1038/s41419-025-07440-3)

Full unedited gel for Figure 1J

β-actin

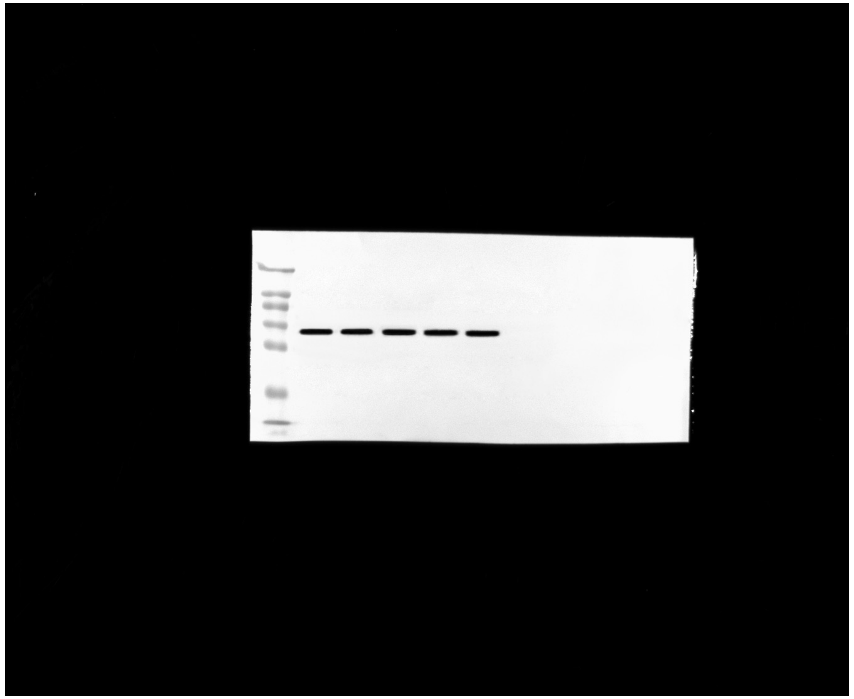

SERPING1

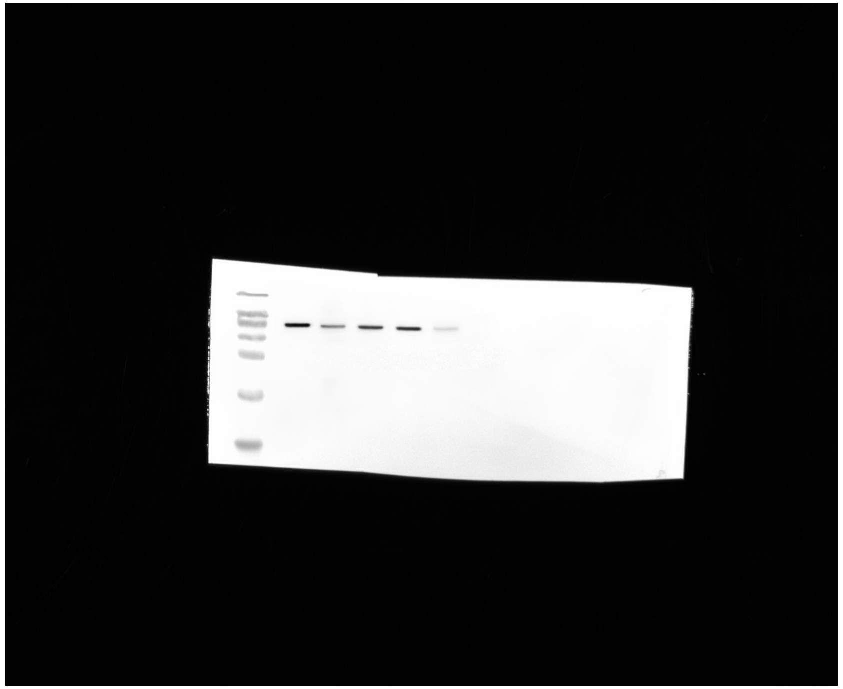

Full unedited gel for Figure 3C

A549

β-actin

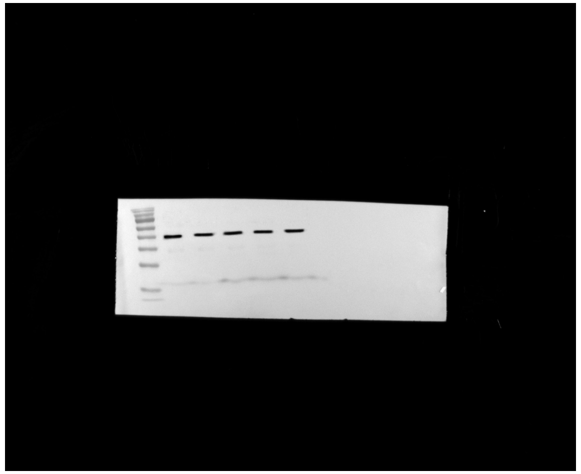

H1299

β-actin

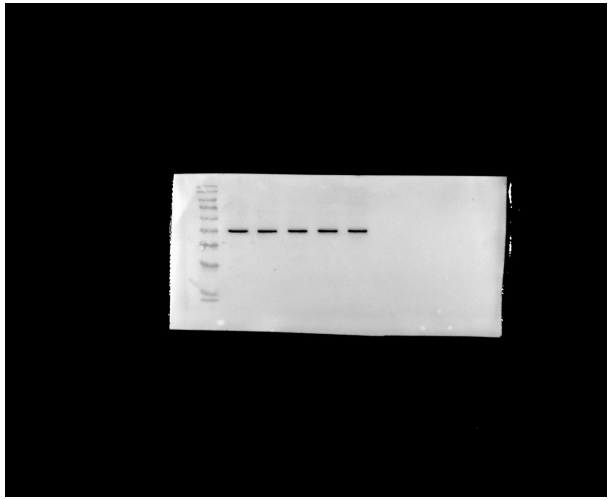

A549

SERPING1

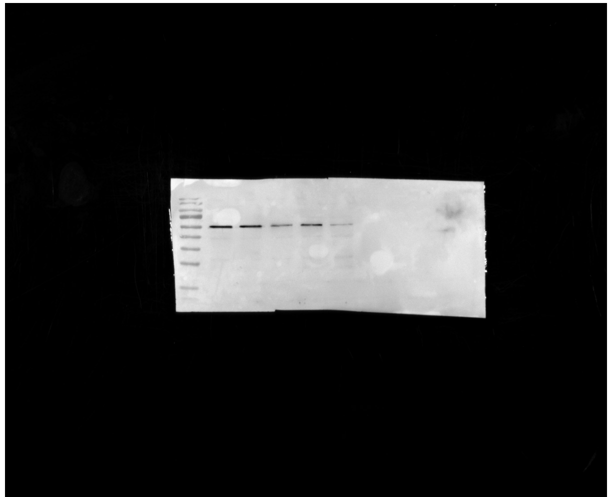

H1299

SERPING1

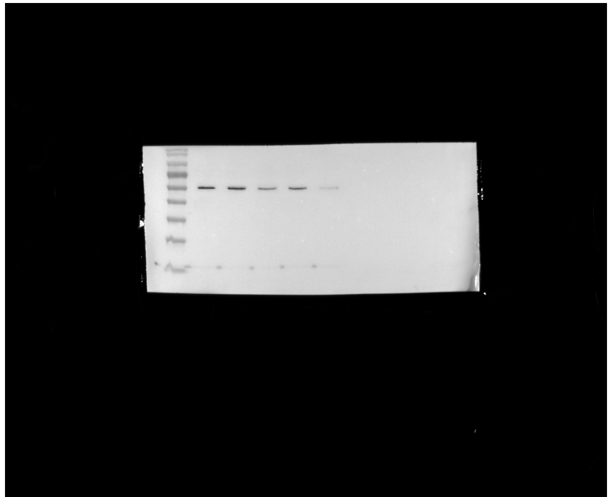

Full unedited gel for Figure 3E

A549

β-actin

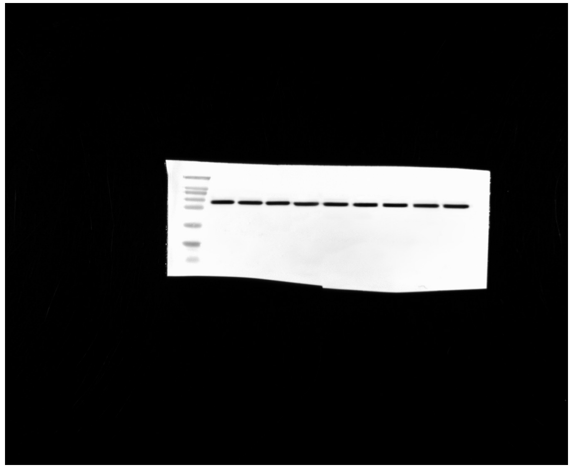

H1299

β-actin

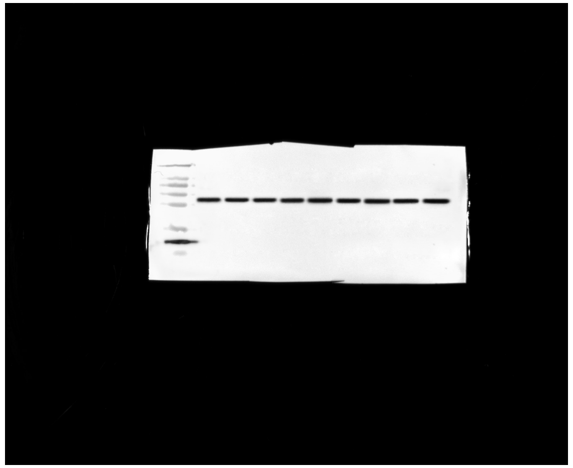

SERPING1

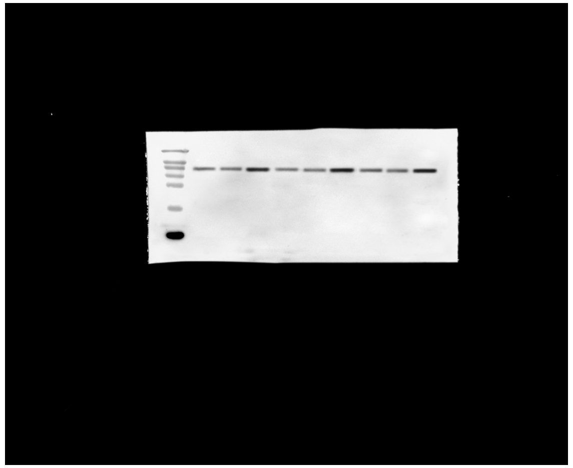

H1299

SERPING1

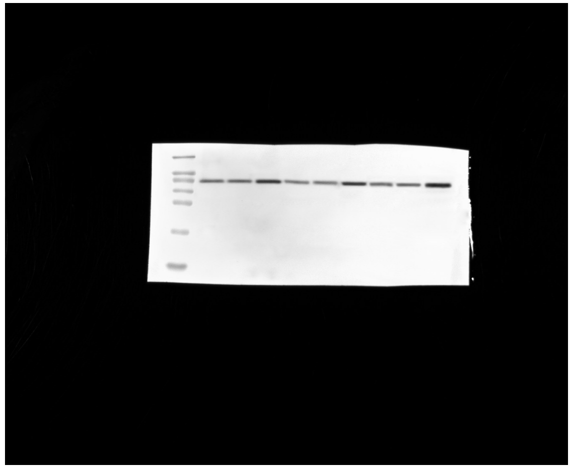

Full unedited gel for Figure 5C

β-actin

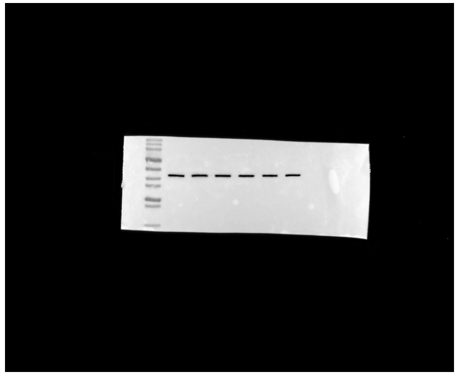

p-mTOR

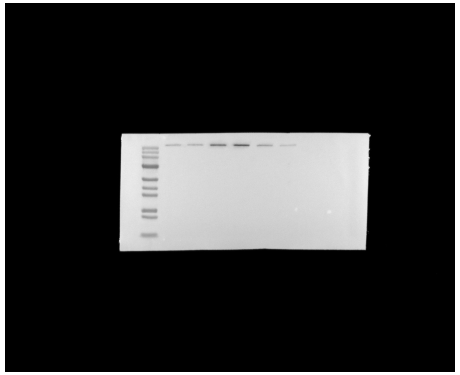

mTOR

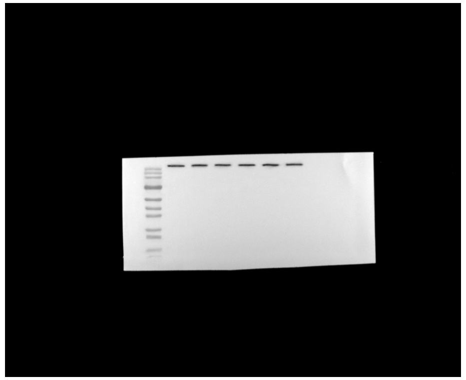

p-4E-BP1

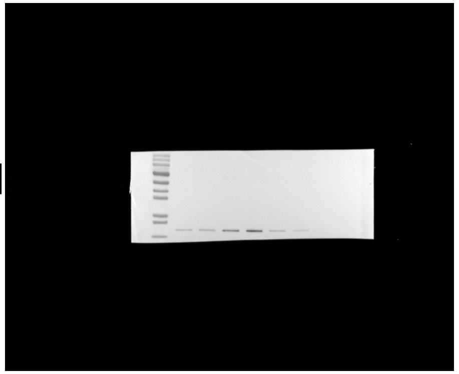

4E-BP1

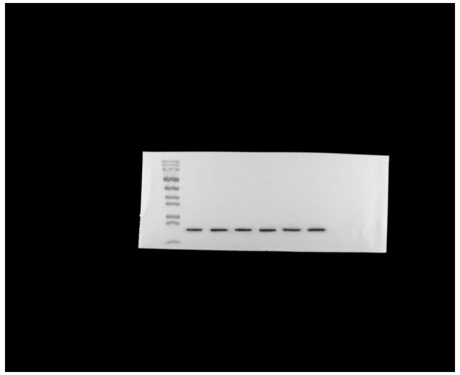

TSC2

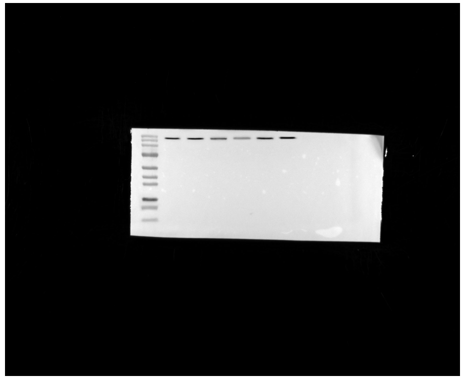

Full unedited gel for Figure 5D

β-actin

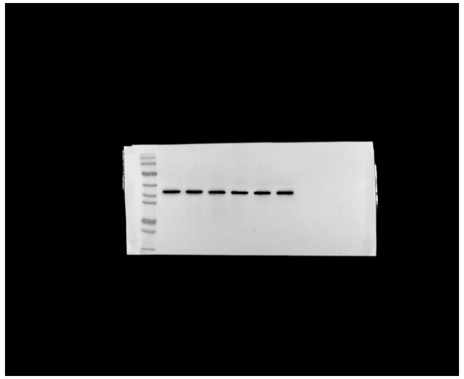

p-mTOR

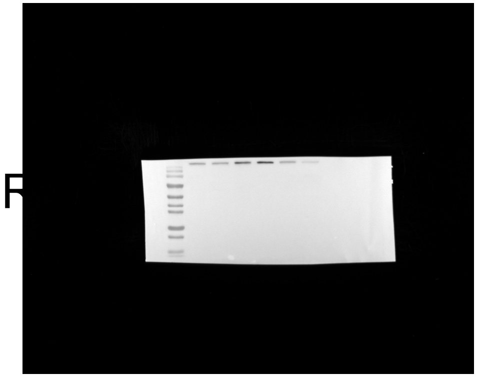

mTOR

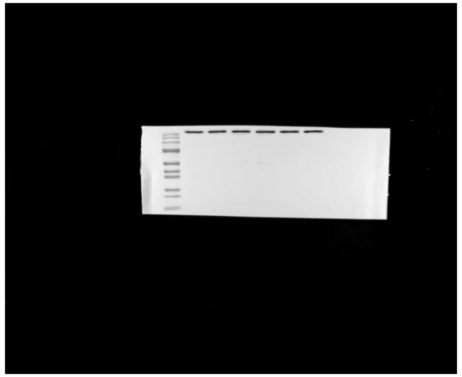

p-4E-BP1

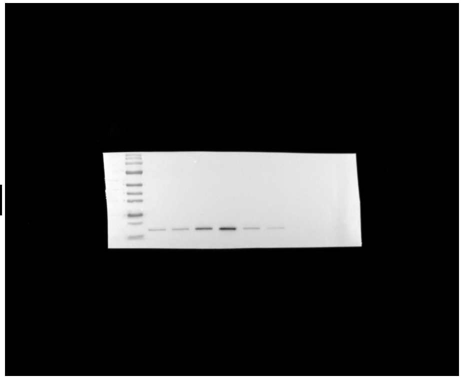

4E-BP1

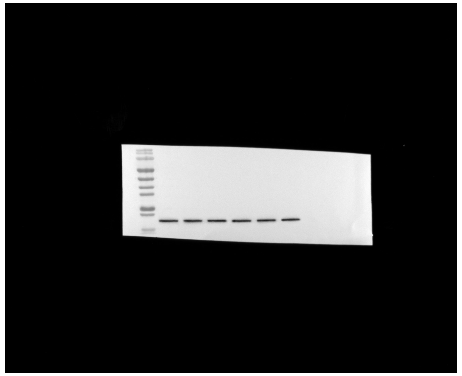

TSC2

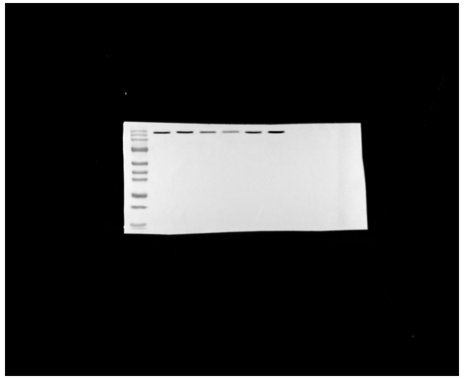

Full unedited gel for Figure 5E

β-actin

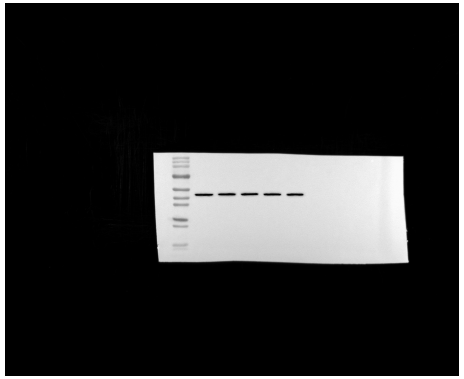

p-mTOR

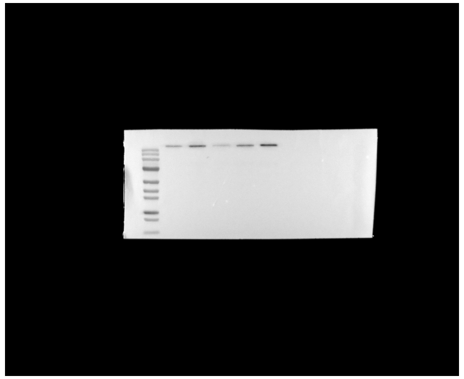

mTOR

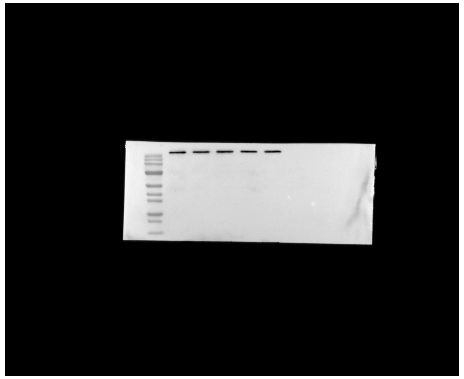

p-4E-BP1

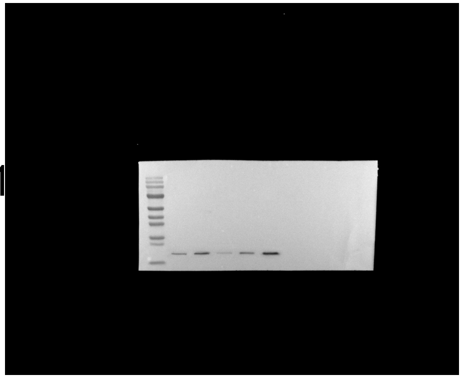

4E-BP1

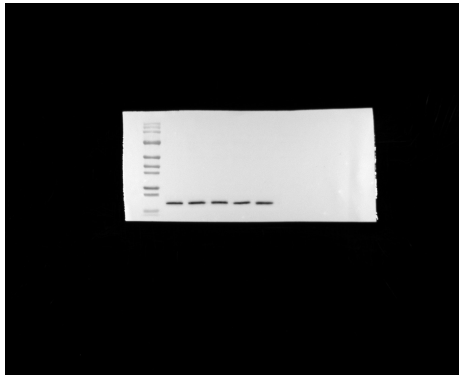

TSC2

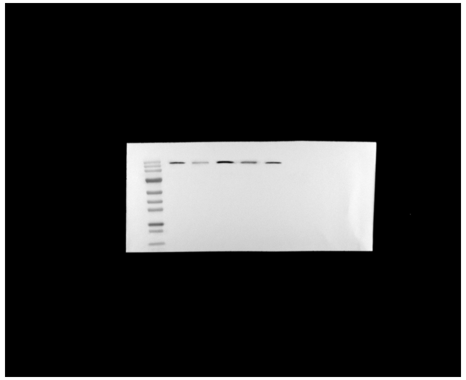

Full unedited gel for Figure 5F

β-actin

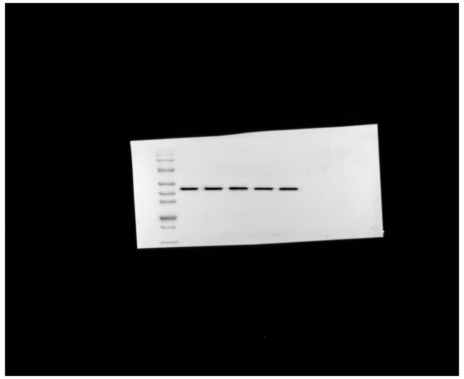

p-mTOR

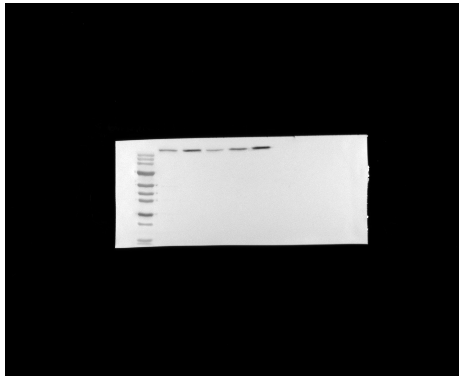

mTOR

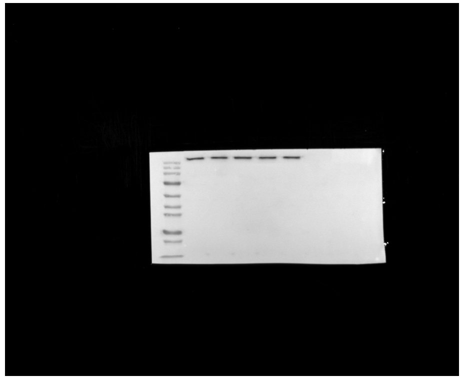

p-4E-BP1

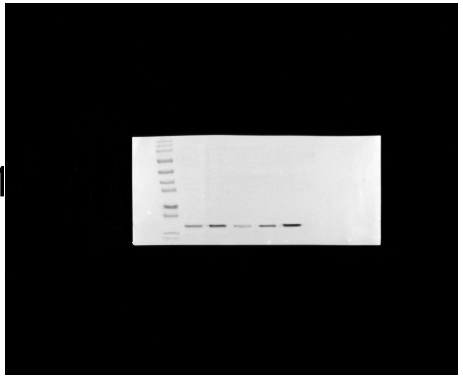

4E-BP1

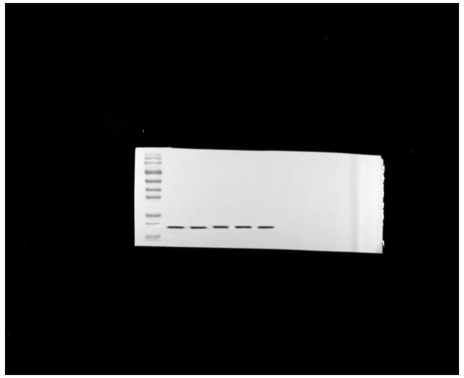

TSC2

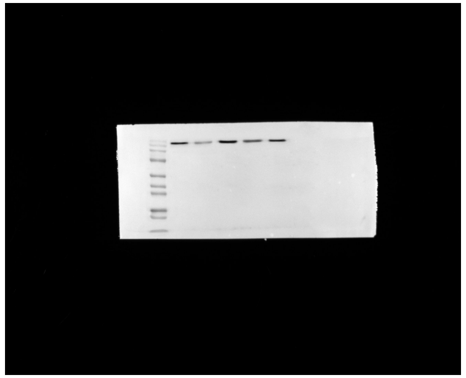

Full unedited gel for Figure 7B

A549

SP5

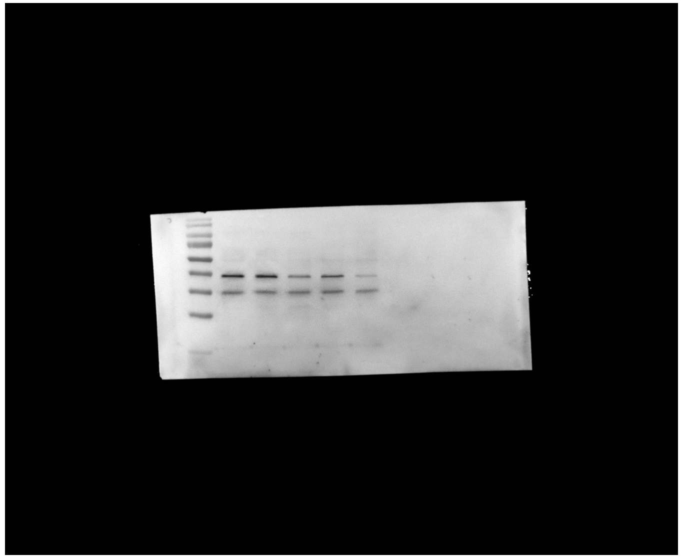

H1299

SP5

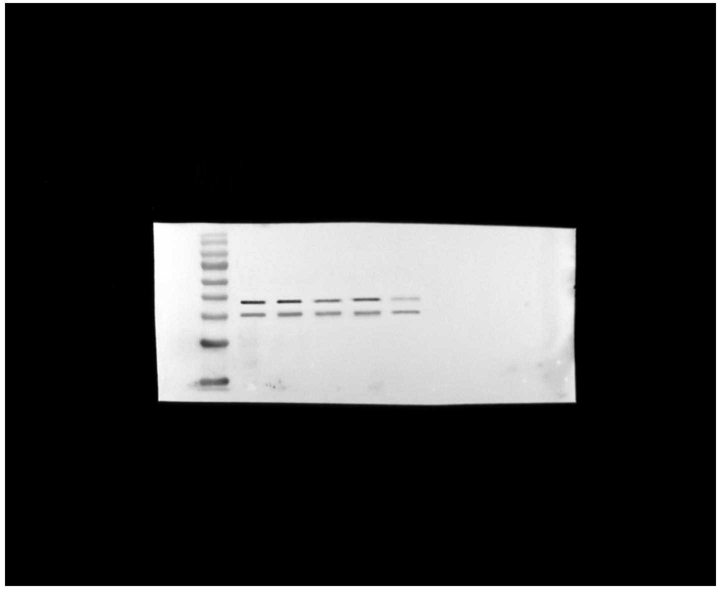

A549

β-actin

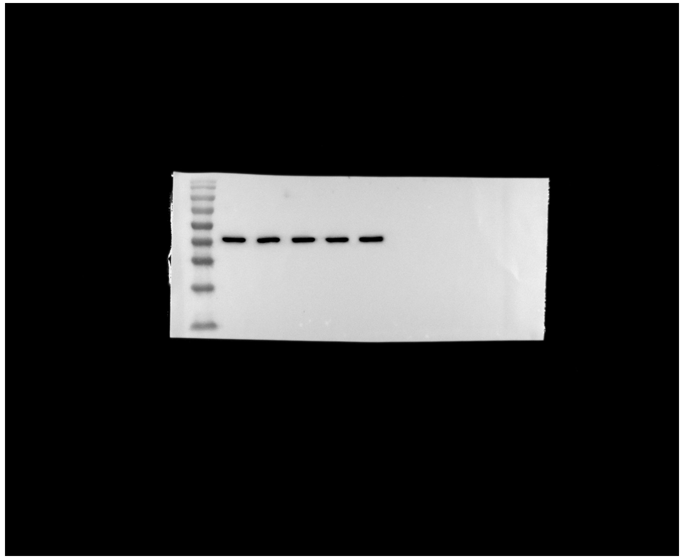

H1299

β-actin

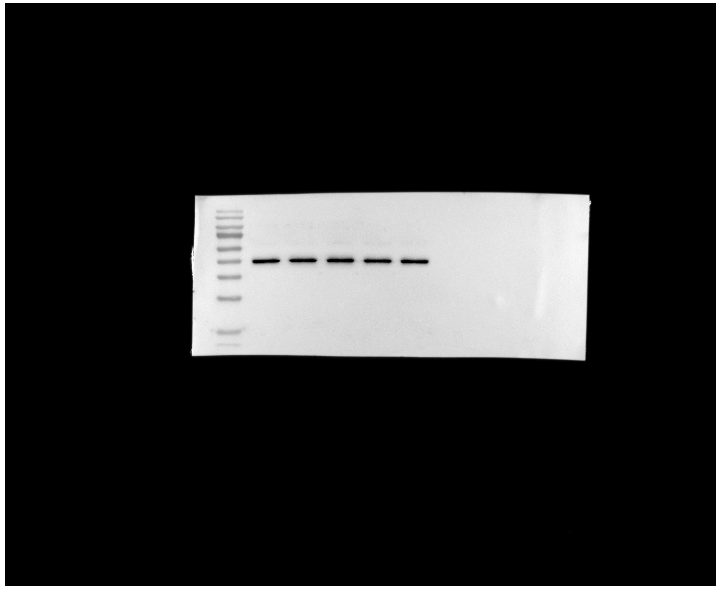

Full unedited gel for Figure 7C

A549

SP5

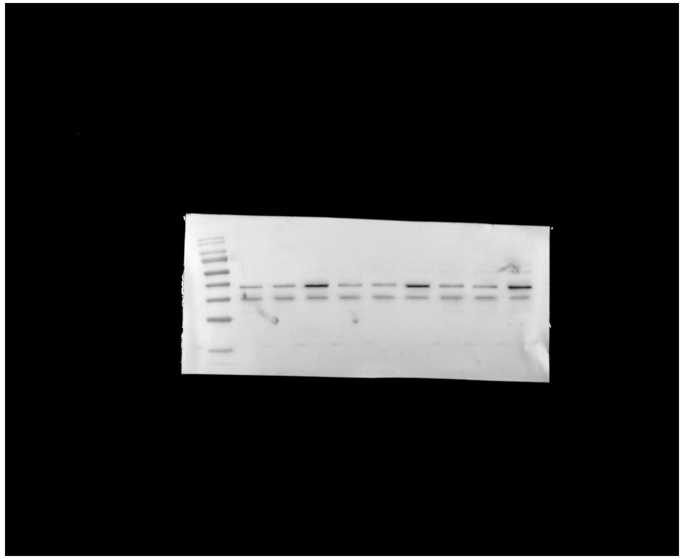

H1299

SP5

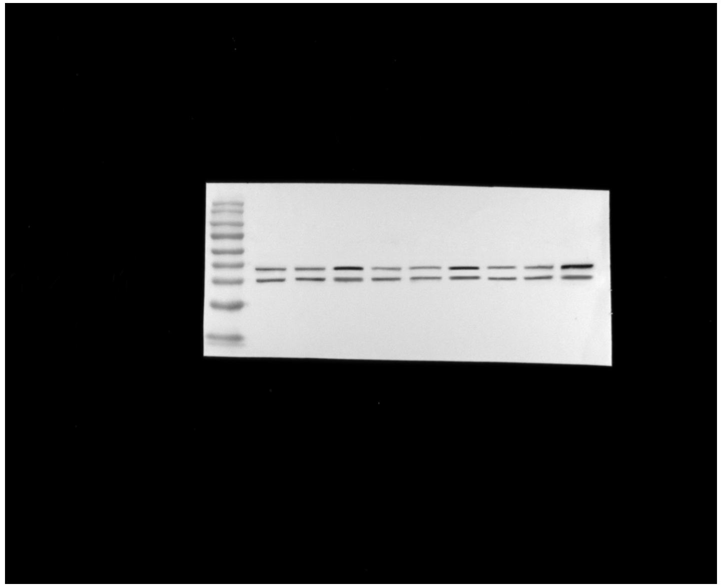

A549

β-actin

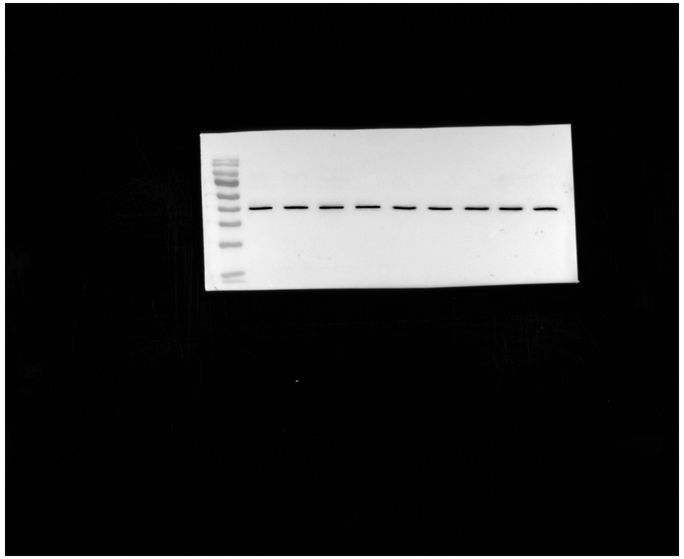

H1299

β-actin

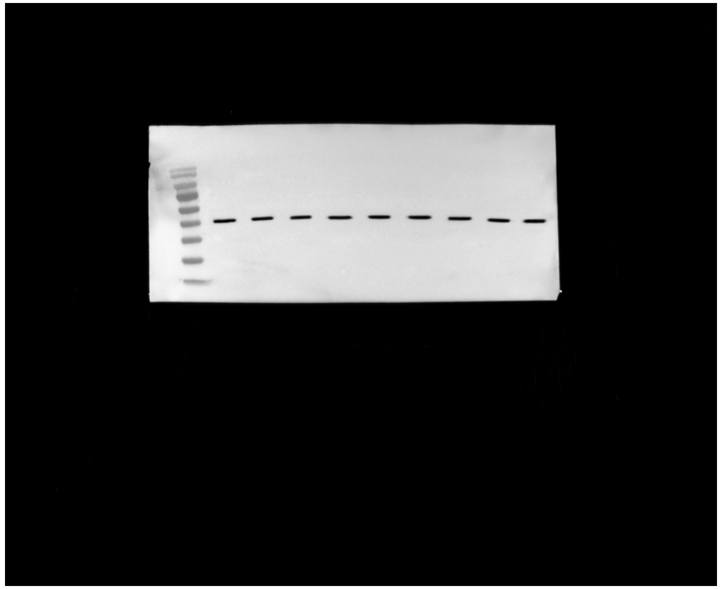

Full unedited gel for Figure 7I

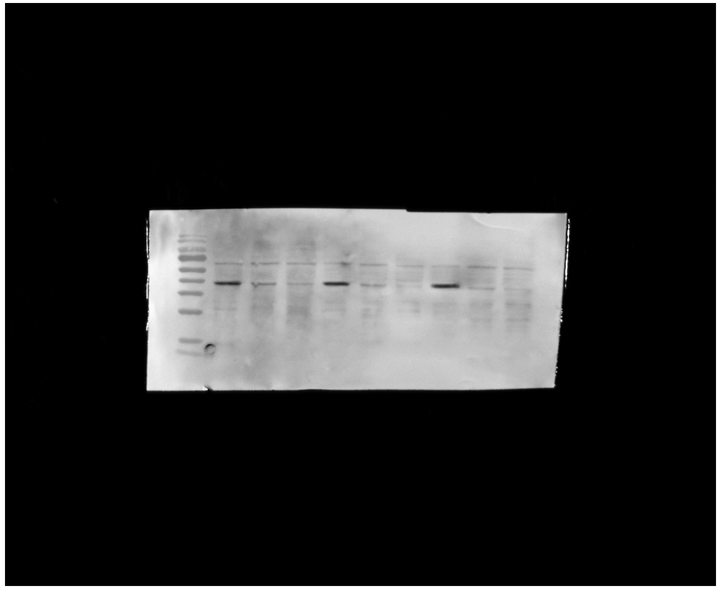

Figure 7I
SP5

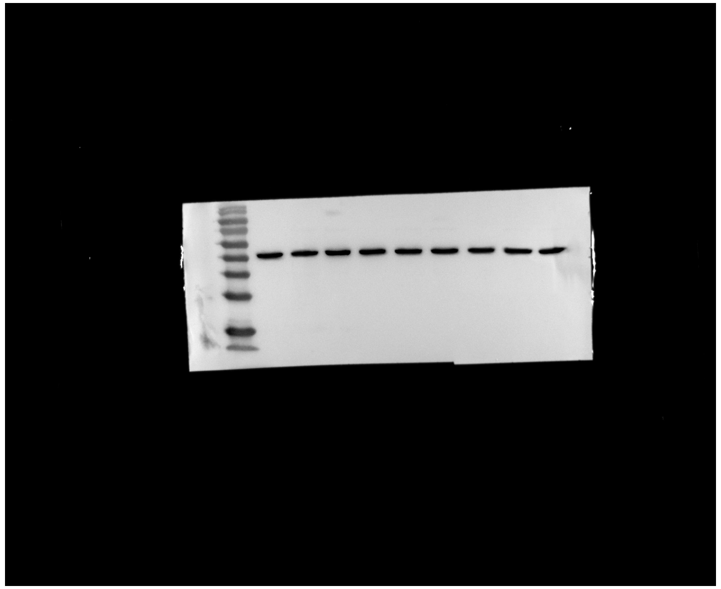

Figure 7I
β-actin

Full unedited gel for Figure S2

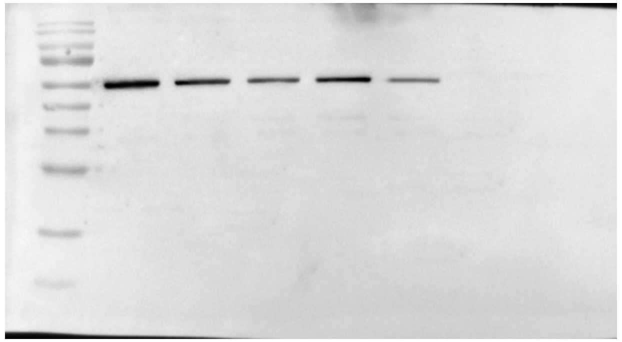

SERPING1

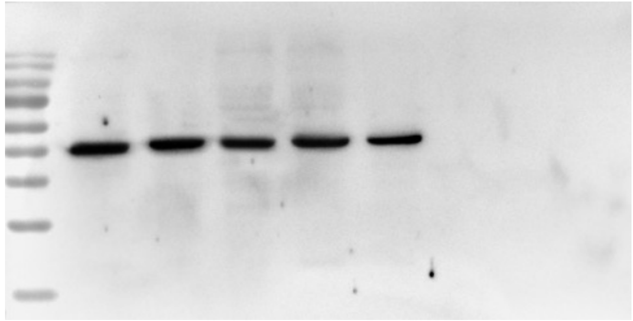

β-actin

Full unedited gel for Figure S3A

β-actin

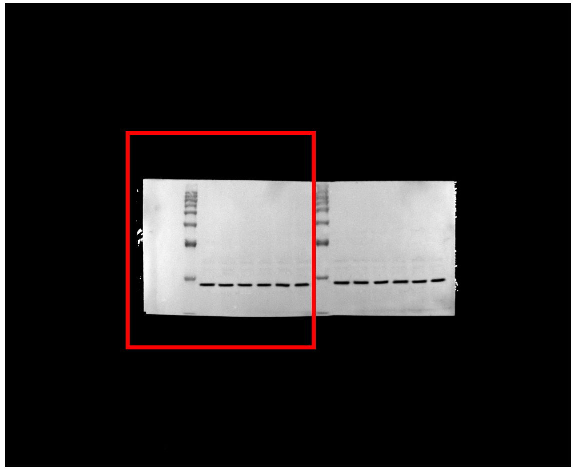

p-mTOR

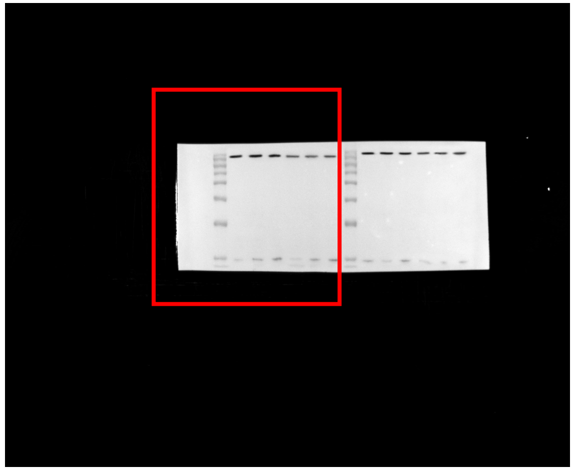

mTOR

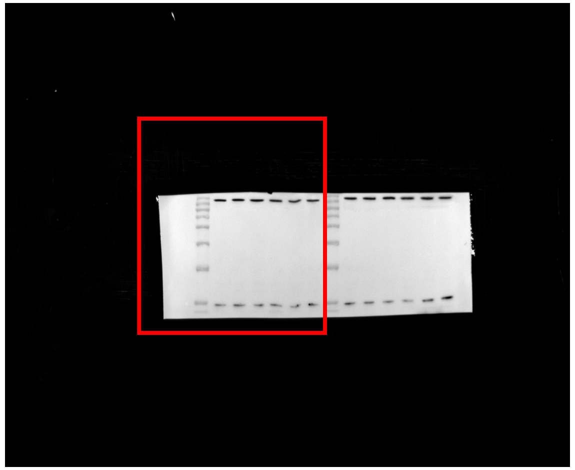

p-4E-BP1

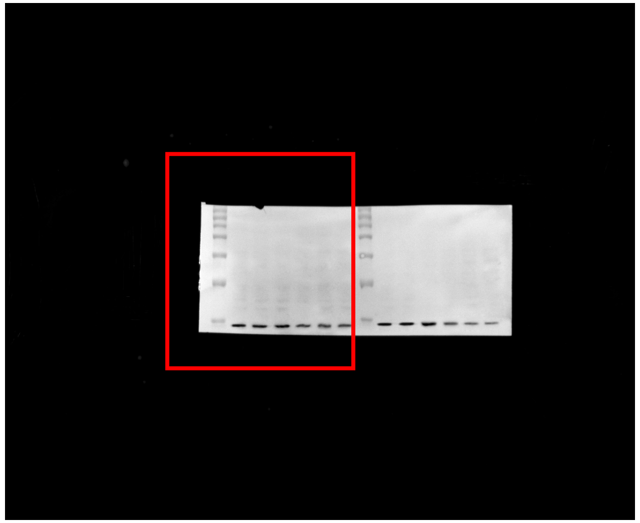

4E-BP1

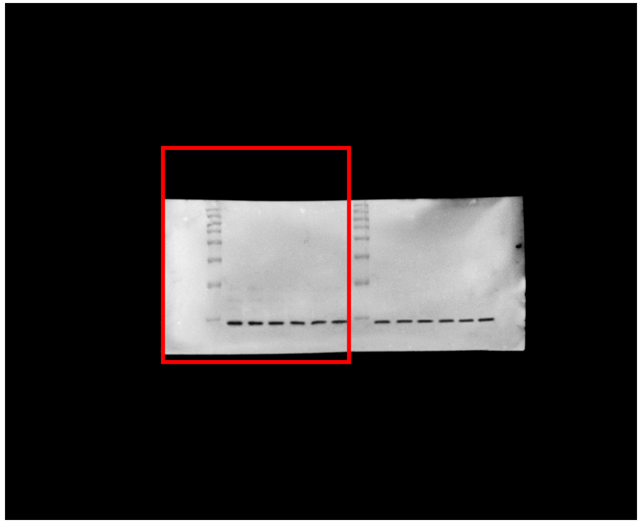

TSC2

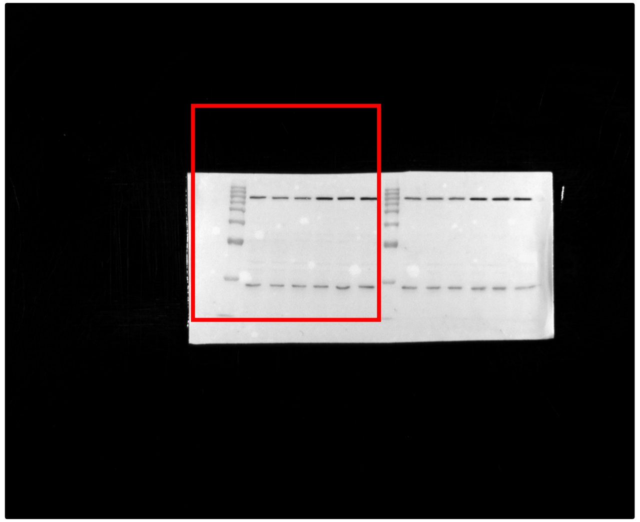

Full unedited gel for Figure S3B

β-actin

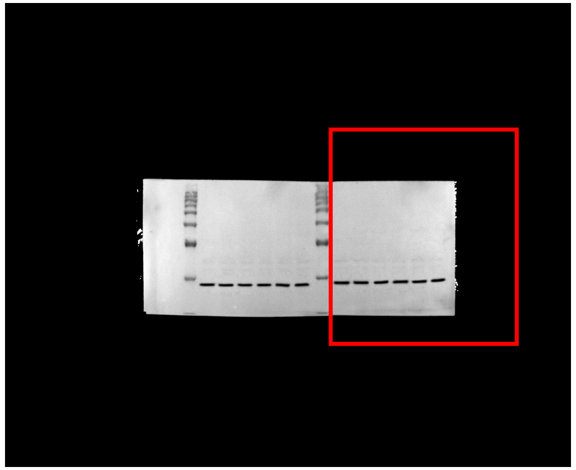

p-mTOR

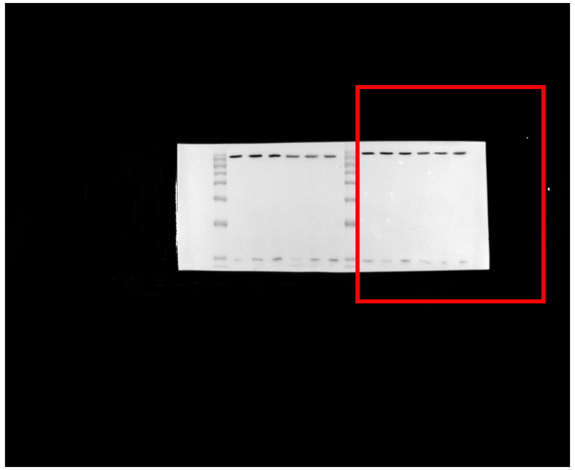

mTOR

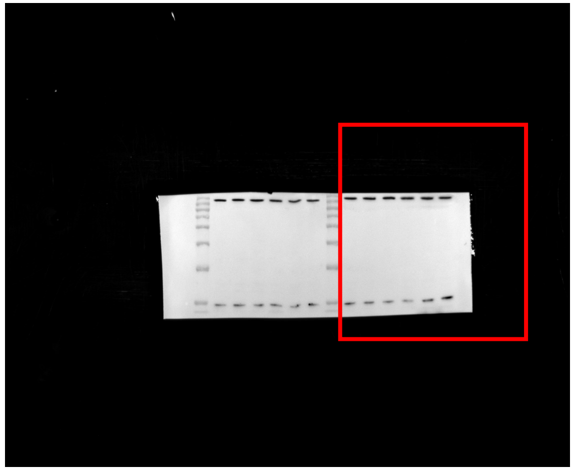

p-4E-BP1

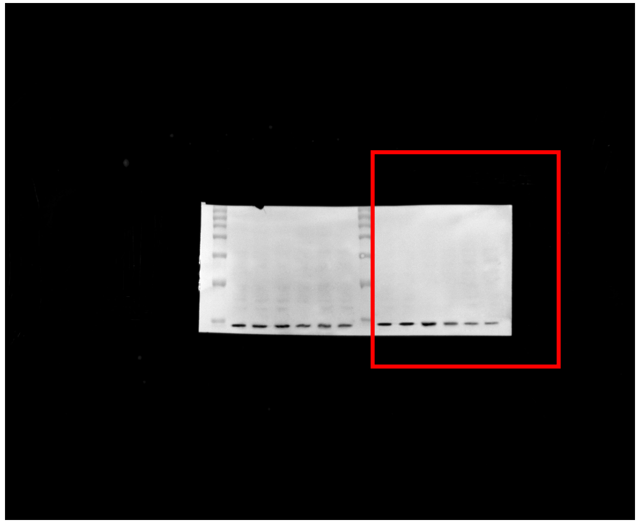

4E-BP1

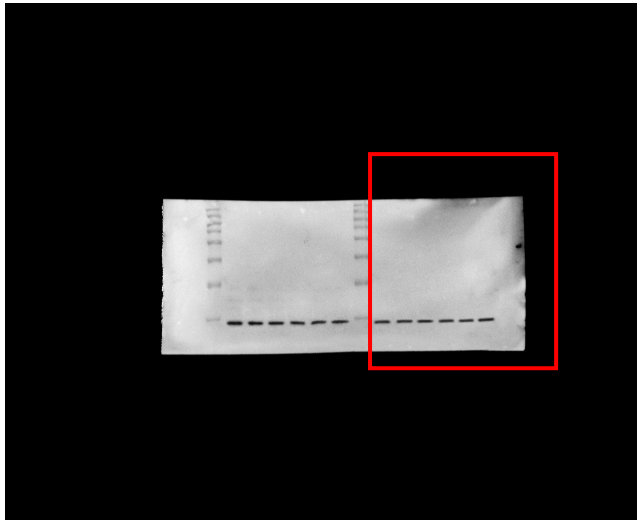

TSC2

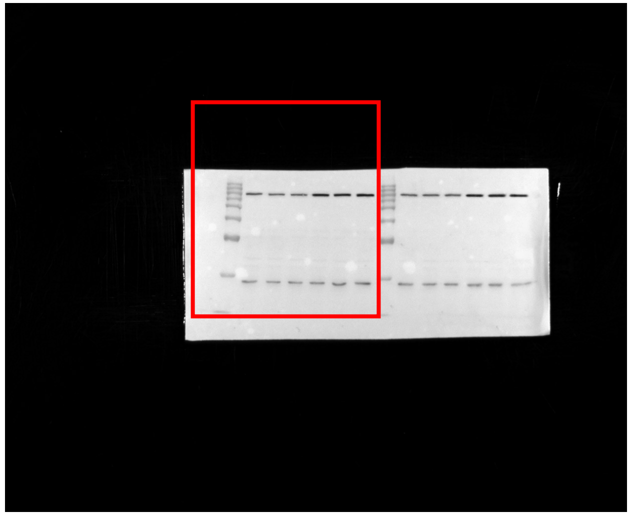

Full unedited gel for Figure S3C

β-actin

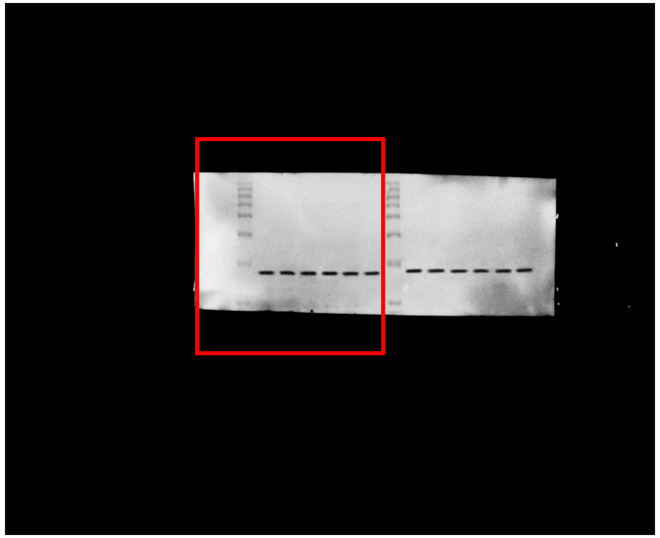

p-mTOR

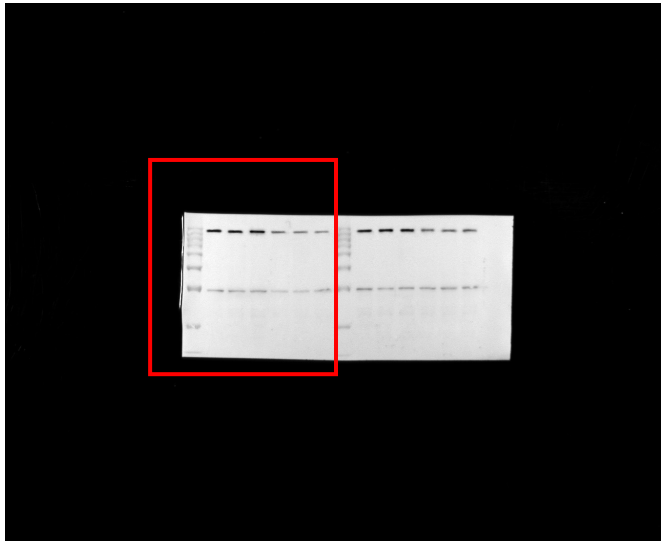

mTOR

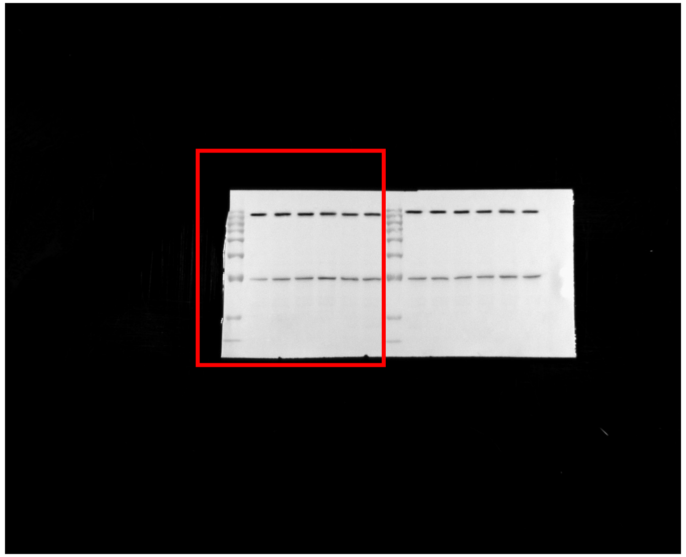

p-4E-BP1

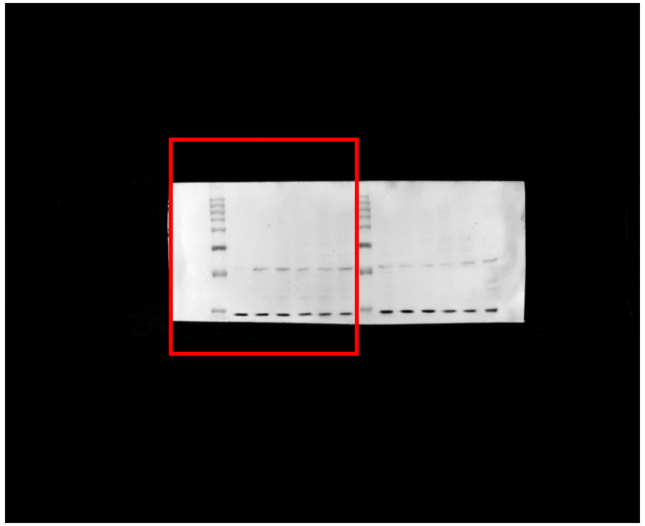

4E-BP1

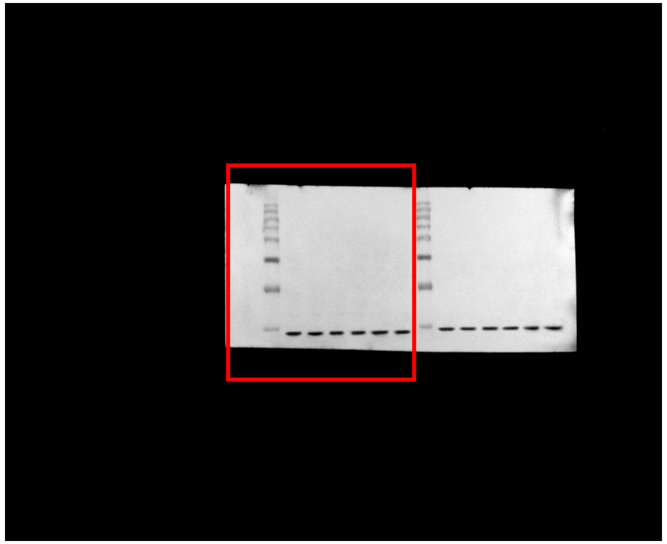

TSC2

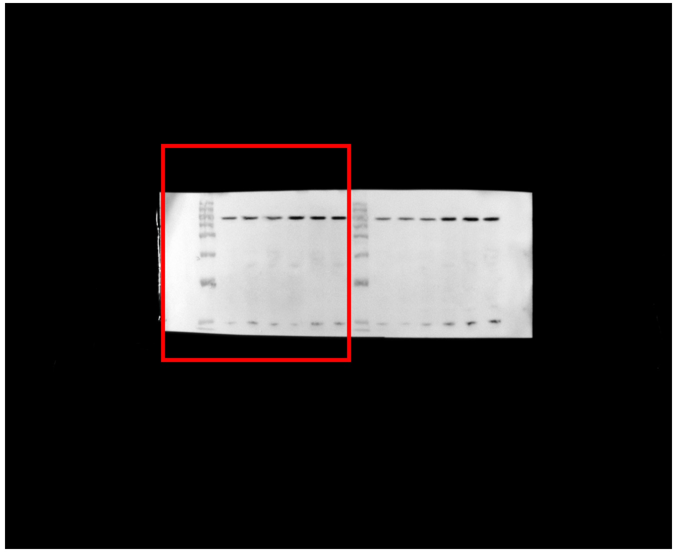

Full unedited gel for Figure S3D

β-actin

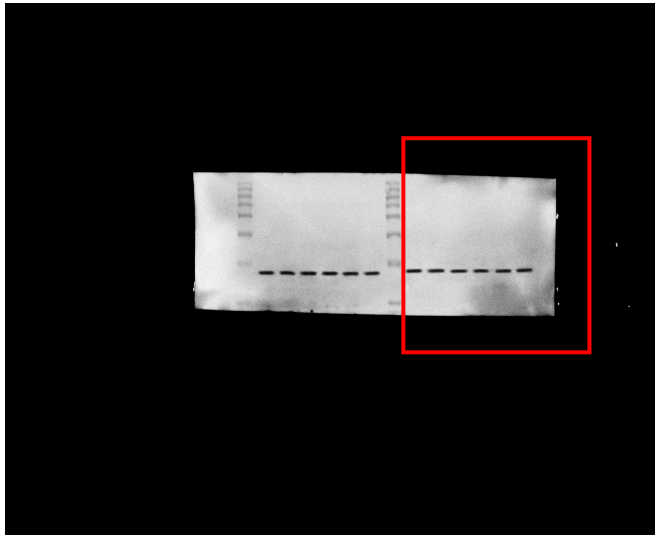

p-mTOR

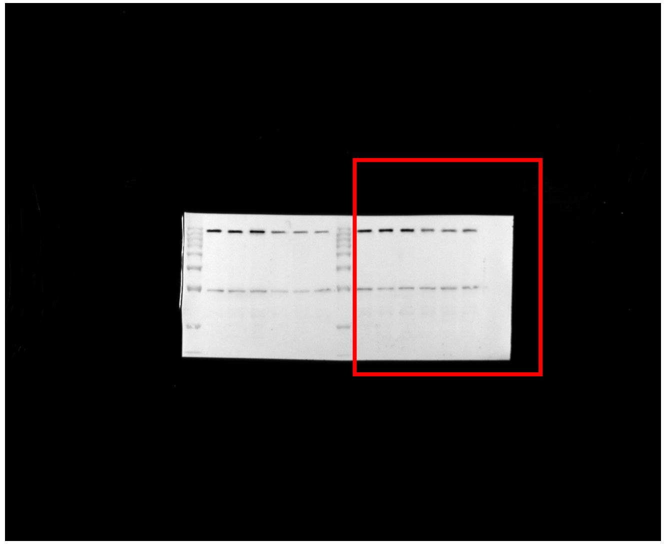

mTOR

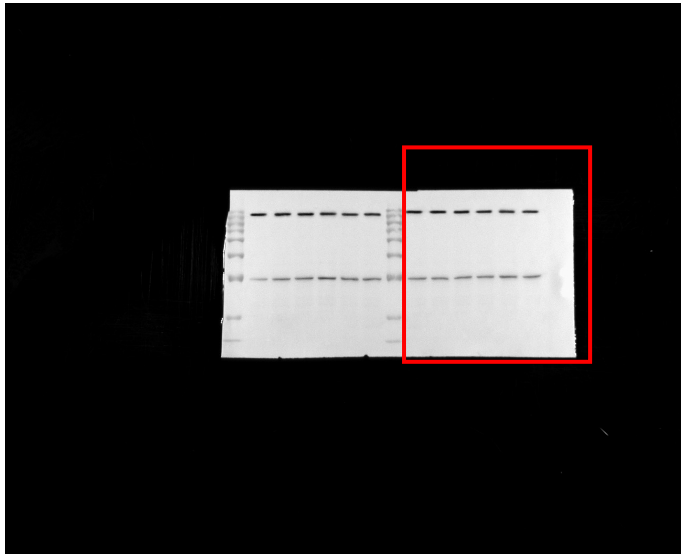

p-4E-BP1

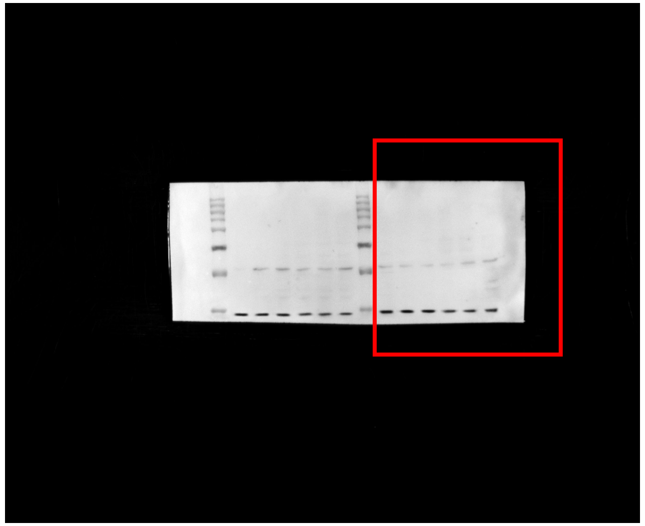

4E-BP1

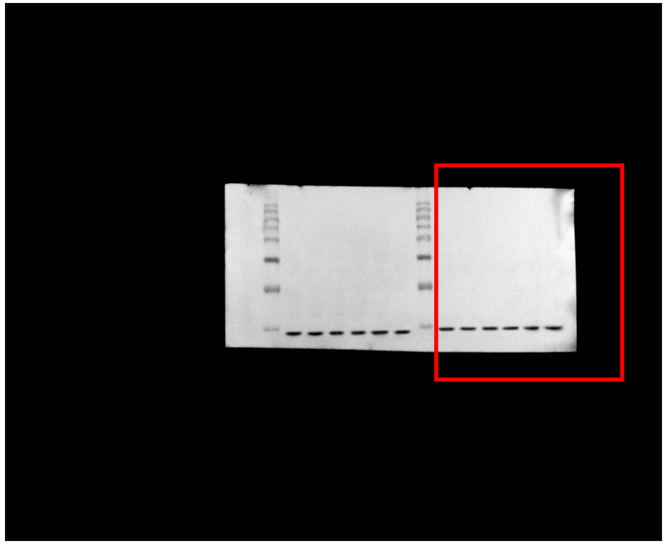

TSC2

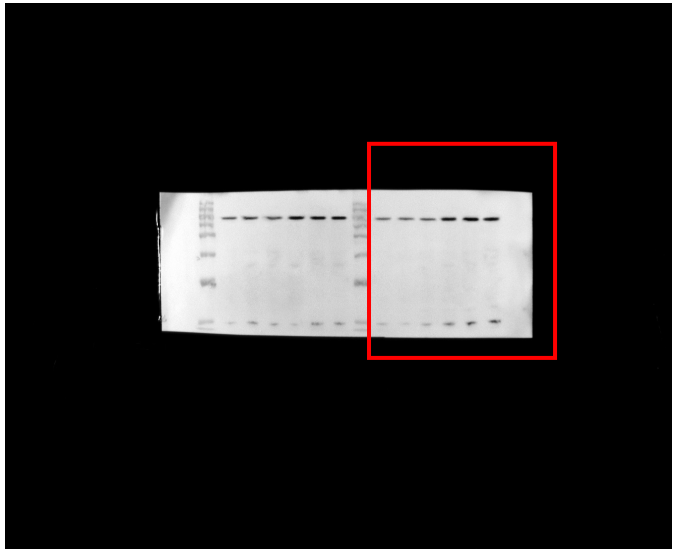

Supplement: Supplementary file 3 — Full unedited gel [file 41419_2025_7440_MOESM3_ESM.pdf]
